# Associations between bioaerosols, lung function work-shift changes and inflammatory markers: A study of recycling workers

**DOI:** 10.5271/sjweh.4187

**Published:** 2024-12-01

**Authors:** Karoline Kærgaard Hansen, Vivi Schlünssen, Karin Broberg, Kirsten Østergaard, Margit W Frederiksen, Torben Sigsgaard, Anne Mette Madsen, Henrik Albert Kolstad

**Affiliations:** 1 Department of Occupational Medicine, Danish Ramazzini Centre, Aarhus University Hospital, DK-8200 Aarhus N, Denmark.; 2 Department of Public Health, Research unit for Environment Occupation and Health, Danish Ramazzini Centre, Aarhus University, DK-8000 Aarhus C, Denmark.; 3 Division of Occupational and Environmental Medicine, Lund University, SE-221 85 Lund, Sweden.; 4 National Research Centre of the Working Environment, DK-2100 Copenhagen Ø, Denmark.; 5 Institute of Clinical Medicine, Aarhus University, DK-8200 Aarhus N, Denmark.

**Keywords:** bacteria, domestic waste, endotoxin, fungi, inhalable dust

## Abstract

**Objectives:**

We investigated associations between bioaerosol exposures and work-shift changes in lung function and inflammatory markers among recycling workers.

**Methods:**

Inhalable dust was measured with personal samplers and analyzed for endotoxin, bacteria, and fungi (incubated at 25 °C and 37 °C) levels. Lung function (FEV1, FVC) was measured before and after work-shifts and serum concentrations of inflammatory markers (CRP, SAA, CC16, IL1B, IL2, IL4, IL5, IL6, IL8, IL10, IL13, and TNF) after the shift. Associations were explored by linear mixed-effects models.

**Results:**

We included 170 measurements from 88 production workers exposed to inhalable dust, endotoxin, bacteria, and fungi (25 °C and 37 °C) at geometric mean levels of 0.6 mg/m^3^, 10.7 EU/m^3^, 1.6×10^4^ CFU/m^3^, 4.4×10^4^ CFU/m^3^, and 10^3^ CFU/m^3^, respectively, and 14 administrative workers exposed at 7-fold lower levels. No associations were observed between bioaerosol exposures and work-shift change in lung function. IL2, IL6, IL10, and TNF concentrations were positively associated with inhalable dust levels, SAA and IL6 with bacteria, CRP, SAA, IL8, and TNF with fungi (25 °C or 37 °C), with the latter being the only statistically significant finding (exp(β) 1.40, 95% confidence interval 1.01–1.96).

**Conclusions:**

This study of recycling workers exposed to bioaerosol levels generally below those of farmers and compost workers and above background levels did not indicate any acute effect on lung function. Several inflammatory markers tended to increase with exposure, suggesting a systemic effect. Future research should combine data from bioaerosol-exposed workers to uncover health risks that may form the basis for health-based occupational exposure limits.

In 2021, a European Union (EU) citizen on average generated 527 kg of domestic waste (waste from the household), of which 31% was recycled (1). The EU has developed a “European Green Deal” in which one of the goals is to increase the recycling of materials (2), and the amount of waste being recycled is anticipated to increase. Over the years, the process of recycling domestic waste has been increasingly automated. However, in Denmark, from 2008 to 2022, the number of employees in the recycling industry increased by 46% (3), and thus it is anticipated in the future these numbers will keep increasing.

Bioaerosols are airborne particles originating from plants, animals, or microorganisms, eg, fungi, bacteria, and endotoxin. Recycling of domestic waste generates bioaerosols that can be inhaled and cause respiratory and systemic health effects for the workers (4, 5). Recycling workers have shown lower lung function compared with administrative workers (6, 7). A few studies of waste workers have found associations between exposure levels of dust, endotoxin, and microorganisms and respiratory symptoms (8). Sigsgaard et al (9) found that increasing exposure to total dust, but not endotoxin, was associated with increasing work-shift decline in forced expiratory volume in the first second (FEV1) among recycling workers. Others have also reported null findings for endotoxin (10, 11).

Bioaerosol components are inflammogenic, and some are cytotoxic (12) but have only been investigated to a limited extent in the recycling industry (13). Total dust and endotoxin were associated with elevated post-shift interleukin (IL)-8 concentrations in nasal lavage fluid among waste collectors, whereas no association was found for IL6, tumor necrosis factor (TNF), or IL1B (14). Among Danish workers recycling biowaste, increasing exposure to anaerobic bacteria was associated with higher serum concentrations of high-sensitivity C-reactive protein (hsCRP) and serum amyloid A (SAA), exposure to fungi incubated at 25 °C was associated with higher concentrations of hsCRP, and exposure to inhalable dust was associated with higher SAA and Uteroglobin (alias Clara cell secretory protein, CC16) (15). However, increasing exposure to endotoxin and bacteria incubated at 25 °C was associated with decreasing concentrations of hsCRP and SAA. A previous study of recycling workers found no clear association between exposure to endotoxin and CRP (10).

In conclusion, studies on the associations between bioaerosol exposures, lung function, and inflammatory markers among recycling workers are few but needed to uncover health risks in this new and growing industry.

We aimed to investigate the associations between exposure to inhalable dust, endotoxin, bacteria, and fungi and work-shift changes in lung function and inflammatory markers among recycling workers.

## Methods

### Study population

This study investigated production workers of recycling plants recorded in the Danish National Waste Register who sorted, shredded, and extracted materials from domestic waste. In Denmark, each municipality is responsible for waste management. Sorting and collection of domestic waste follow the National Plan for Prevention and Management of Waste 2020-2032. According to this, waste must be separated into ten different fractions by each household before further processing (16, 17). After collection, the waste is further sorted (manually or mechanically), shredded, and extracted at different facilities. In Denmark, extracting facilities mainly handle plastic waste, as the other fractions are often exported to larger facilities outside Denmark. As a reference population, administrative workers from the participating plants were recruited. Both production and administrative workers volunteered to participate. The industry is heavily male-dominated and therefore only men were recruited.

We recently found that the production workers were 7-fold or higher exposed to inhalable dust, endotoxin, bacteria, and fungi than the administrative workers (18). The geometric mean (GM) exposure levels of inhalable dust, endotoxin, bacteria, and fungi (incubated at 25 °C and 37 °C) among production workers were 0.6 mg/m^3^ [(geometric standard deviation (GSD) = 3.7], 10.7 EU/m^3^ (4.2), 1.6×10^4^ CFU/m^3^ (4.5), 4.4×10^4^ CFU/m^3^ (8.7), and 10^3^ CFU/m^3^ (5.1), respectively. The corresponding exposure levels among the administrative workers were 0.0 mg/m^3^ (2.1), 1.3 EU/m^3^ (2.3), 2.2 × 10^3^ CFU/m^3^ (4.0), 5.5 × 10^2^ CFU/m^3^ (2.3), and 10^2^ CFU/m^3^ (4.6), respectively.

A detailed description of the study population, exposure assessment, and classification of the production characteristics is found elsewhere (18).

The study was registered at the repository of the Central Denmark Region (1-16-02-715-20), and the Central Denmark Region Committees on Health Research Ethics approved the study (1-10-72-322-20). All participants gave informed consent and could withdraw from the study at any time point.

### Data collection

Data was collected on Tuesdays, Wednesdays, or Thursdays in April through July 2021 and again in August through October 2021. Participants thus contributed with up to two measurements each to take season variations in exposure levels into account.

### Outcome assessment

Lung function was measured pre-shift (morning measurement) and post-shift (afternoon measurement) with dynamic spirometry using an NDD EasyOne Air Spirometer. Exclusion criteria for performing lung function were recent heart problems; thoracic, eye, or abdominal surgery; corneal detachment, or pneumonia. At least two persons independently evaluated the quality of all blows according to modified American Thoracic Society (ATS) and European Respiratory Society (ERS) criteria (19, 20). The outcomes were work-shift change in absolute values of FEV1, forced vital capacity (FVC), and FEV1/FVC. The change in these parameters was calculated as the difference between afternoon and morning values.

Blood samples (BD Vacutainer SST blood collection tubes, 10 ml) were collected at the end of the shift, separated by centrifugation, and serum was stored at -80 °C until analysis. We measured the following inflammatory markers (minimum detectable dose, MDD): IL1B (0.25 pg/mL), IL2 (0.25 pg/ml), IL4 (0.07 pg/ml), IL5 (0.31 pg/ml), IL6 (0.38 pg/ml), IL8 (0.36 pg/ml), IL10 (5.47 pg/ml), IL13 (3.39 pg/ml), and TNF (0.62 pg/ml) in serum with Human XL Cytokine Luminex Performance Assays (R&D Systems, Inc. Minneapolis, MN); CRP (0.01 ng/ml) in serum with Quantikine ELISA (R&D Systems, Inc. Minneapolis, MN); SAA (4 ng/ml) in serum with Human SAA ELISA Kit (Thermo Fisher Scientific Inc., Waltham, MA); and CC16 (0.07 ng/ml) in serum with Quantikine ELISA (R&D Systems, Inc. Minneapolis, MN). The samples were randomized in 96-well plates and standard curves were included with each plate. For the Human XL Cytokine Luminex Performance Assays, 9 standard curves were run for each plate.

### Exposure assessment

Full shift inhalable dust was collected on a 37-mm glass fiber (GFA) filter (Whatman International Ltd, Maidstone, UK) with personal conductive plastic conical inhalable sampler (CIS) cartridges (JS Holdings, Stevenage, UK) connected to SKC AirChek XR5000 portable pump (SKC Inc., Eighty-Four, PA) with a flow rate of 3.5 l/min. The cassette was placed on the upper part of the chest. Inhalable dust was determined gravimetrically, and the filters were stored for a minimum of 24 hours at 22 °C and 45% relative humidity before weighing using a Mettler UMT2 analytical scale (Mettler-Toledo Ltd, Greifensee, Switzerland) with 0.1 mg precision. For each measurement day, one field blank was included (N=24). The lower limit of detection (LOD) for inhalable dust was calculated as three times the standard deviation (SD) of the mean of the field blanks, corresponding to a LOD of 14 µg/m^3^.

Endotoxin was extracted from the collected inhalable dust samples and thus analyzed on all participants. Endotoxin was measured by the Limulus Amoebocyte Lysate (LAL) test (Kinetic-QCL 50-650U kit, Lonza, Walkersville, Maryland, USA). The extraction was performed in 5 ml of pyrogen-free water with 0.05% (v/v) Tween-20. A standard curve based on *Escherichia coli* O55:B5 reference endotoxin was used to quantify the exposure levels as endotoxin units (EU) (21). The analytical level of quantification (LOQ) was 0.05 EU/m^3^.

Microorganisms were sampled among participants who reported that they worked with waste fractions that had been in contact with organic material and all the administrative workers from the companies employing these workers. Microorganisms were sampled in parallel with inhalable dust on separate 37-mm polycarbonate filters and pumps, and extracted in MilliQ water with 0.85% NaCl and 0.05% Tween80 with 33% glycerol and stored at -80 °C. For quantification of bacteria and fungi, a representative dilution for each sample with optimal coverage and separation of individual CFUs was chosen. Dust suspensions were then plated on Dichloran Glycerol agar (DG-18 agar; Thermo Fisher Scientific Oxoid, Basingstoke, UK) and incubated at 25 °C and 37 °C (to encourage the growth of human pathogens) for seven and three days, respectively for quantification of fungi. Dust suspensions were also plated on Nutrient agar (NA; Thermo Fisher Scientific Oxoid, Basingstoke, UK) plates with actidione (cycloheximide; 50 mg/l; Serva, Germany) and incubated at 25 °C for seven days, and on Fastidious Anaerobe Agar (FAA) and incubated anaerobic for 40 h at 37 °C for quantification of bacteria (15). The LOD was 42 CFU fungi and bacteria/m^3^ based on the sampled volumes, the extraction volume, and the plated amounts.

### Participant and production characteristics

During the measurement day, we observed and interviewed each participant about primary work task, waste fractions, work location (indoor or outdoor), filtered ventilation of vehicles or indoor work location, and use of personal protective equipment (PPE). We also asked about age, smoking, asthma, and allergy status, and measured height and weight.

### Statistical analysis

The analytical unit was measurement and each participant contributed with up to two measurements each. Exposure levels below LOD or LOQ were assigned a value of LOD/2 or LOQ/2 (22). To identify correlations between exposures, Pearson pairwise correlation coefficients were calculated for all exposures.

The results of the unadjusted analyses were presented graphically as boxplots. Results indicating associations, independent of statistical significance and the value of the β-estimate, were included in the manuscript. All boxplots are shown in the supplementary material. We analyzed the associations with lung function using linear mixed effects models and analyzed inflammatory markers with linear mixed effects Tobit models (metobit, Stata) for interval-censored data because most inflammatory markers included concentrations below MDD. Inflammatory marker concentrations below MDD were assumed to be in an interval between (-∞) and the MDD (23, 24). Inflammatory marker concentrations were right-skewed; hence, they were natural log-transformed. All exposures were analyzed categorically by quartiles and continuously by a log10-transformed variable to linearize the relationship. All models included time of measurement (morning and afternoon), season (spring, summer, and autumn), smoking (current, former, and never smoker), self-reported current allergy (yes and no), self-reported current asthma (yes and no), body mass index (BMI) (<25, 25–30, and ≥30 kg/m^2^), and age (years, continuous) as fixed effects and worker as random effect. These covariates were decided a priori as they previously are shown to predict lung function and inflammatory marker levels (25–27). We also assessed if season modified the results by adding an interaction term of season and the respective exposures.

In the analysis of work-shift change in lung function, the β-coefficients of the categorical exposure models express the absolute difference compared with the lowest quartile, and the β-coefficients of the continuous exposure models express the absolute difference per 10-fold increase in exposure.

In the analysis of inflammatory markers, the exp(β)-coefficients of the categorical exposure models express the ratio between means compared with the lowest quartile, and the exp(β)-coefficients of the continuous exposure models express the ratio between means per 10-fold increase in exposure.

In supplementary analyses of production characteristics, lung function, and inflammatory markers, all production characteristics were categorical, and thus the β-coefficients express the absolute or ratio between means compared with the reference.

All analyses were carried out using Stata, version 17 (StataCorp, College Station, TX, USA).

## Results

**Figure 1:**
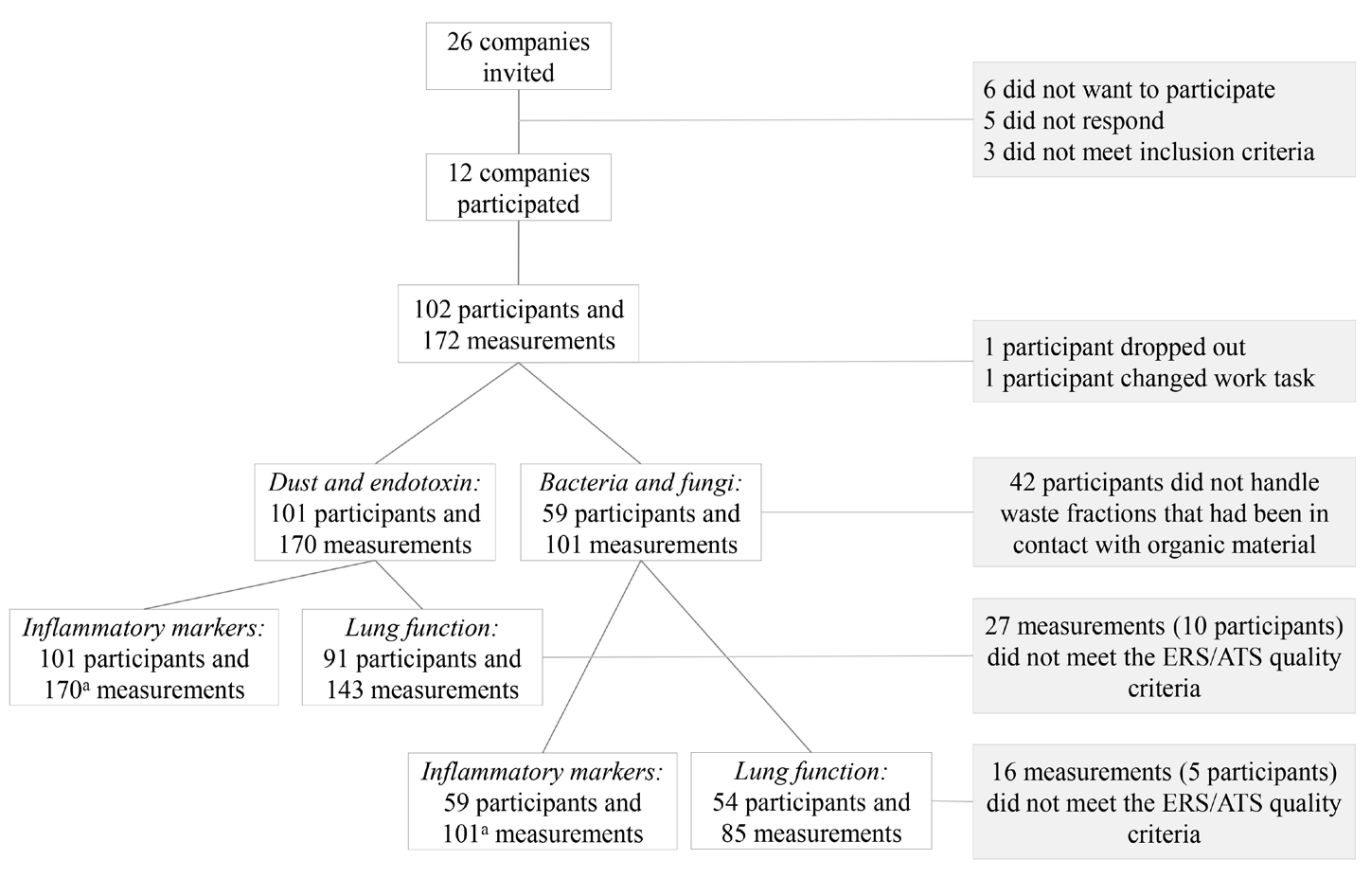
Flow chart.

In total, 12 out of 26 invited companies agreed to participate (46%) of which 8 companies sorted domestic waste, 2 sorted and shredded domestic waste, and 2 shredded and extracted raw materials from plastic waste. In total, 102 participants provided 172 measurements. There were 101 participants and 170 measurements for further analysis after one participant and two measurements were discarded (drop-out during measurement and change of work task to exaggerate dust levels). The analyses of bacteria and fungi included 59 participants (101 measurements, 7 companies) (figure 1).

All participants met the inclusion criteria for lung function testing, but 27 of the measurements did not meet the ERS/ATS quality criteria, leaving 143 measurements from 91 participants for further analyses (figure 1). We analyzed 170 blood samples from 101 participants for inflammatory markers. For one sample, we only had enough serum to measure CRP, SAA, and CC16. For IL4, IL5, and IL13 >75% of the measurements were below MDD (76%, 86%, and 82%, respectively), and they were not included in the statistical analysis.

Production workers tended to have higher BMI and were more frequent current smokers than the administrative workers, but otherwise, the two groups showed similar characteristics (table 1). In line with GDPR regulations, only participant and measurement characteristics are shown in table 1 if observations are ≥5.

**Table 1:** Characteristics of 170 measurements by participant and measurement characteristics among 88 production workers and 14 administrative workers.^a^

Characteristics		Production workers			Administrative workers	
		N (%)	Mean (IQR) ^b^		N (%)	Mean (IQR) ^b^
Age			49 (42-60)			50 (44-58)
Body mass index (kg/m^2^)						
	<25	28 (19)			≤5 ^a^	
	25–30	48 (32)			16 (76)	
	≥30	73 (49)			≤5 ^a^	
Smoking status						
	Current	47 (31)			≤5 ^a^	
	Former	37 (25)			≤10 ^a^	
	Never	65 (44)			12 (57)	
Current asthma						
	Yes	14 (9)			≤5 ^a^	
	No	135 (91)			≤20 ^a^	
Current allergy						
	Yes	29 (19)			≤5 ^a^	
	No	120 (81)			≤20 ^a^	
Season						
	Spring (April, May)	32 (22)			6 (29)	
	Summer (June, July, August)	57 (38)			7 (33)	
	Autumn (September, October)	60 (40)			8 (38)	
Time of morning measurement			7 (7–8)			8 (8–8)
Time of afternoon measurement			14 (14–15)			13 (12–15)

^a^ In line with GDPR regulations, only participant and measurement characteristics for production workers are shown, due to few (<5) observations for some of the characteristics among the administrative workers. ^b^ Interquartile range (IQR) shown as 25% percentile and 75% percentile.

### Associations between bioaerosol exposure levels and work-shift change in lung function

We observed a decline in FVC for the upper quartile of fungal exposure level, but no overall associations between the bioaerosol exposure levels and work-shift change in FEV1, FVC, or FEV1/FVC (table 2, supplementary material, www.sjweh.fi/article/4187, figures S1-S5).

**Table 2:** Absolute adjusted work-shift change in lung function by bioaerosols exposure levels among recycling workers.^a^

Bioaerosols		Participants	Samples	Change in FEV1 (L/min)		Change in FVC (L/min)		Change in FEV1/FVC
				β (95% CI)		β (95% CI)		β (95% CI)
Inhalable dust (mg/m^3^)								
	<0.1	28	37	Reference		Reference		Reference
	0.2–0.3	36	39	-0.04 (-0.21–0.12)		-0.06 (-0.20–0.08)		0.00 (-0.03–0.03)
	0.4–1.0	30	33	0.04 (-0.14–0.22)		0.08 (-0.07–0.23)		-0.01 (-0.03–0.02)
	>1.1	27	34	0.09 (-0.10–0.27)		0.01 (-0.15–0.16)		0.01 (-0.02–0.04)
	Per 10-fold increase in exposure	91	143	0.01 (-0.10–0.13)		-0.03 (-0.12–0.07)		0.00 (-0.01–0.02)
Endotoxin (EU/m^3^)								
	<2.5	28	35	Reference		Reference		Reference
	2.6–6.4	35	37	0.07 (-0.10–0.24)		0.07 (-0.07–0.22)		0.00 (-0.03–0.03)
	6.5–21.4	34	38	0.16 (-0.02–0.33)		0.16 (0.01–0.31)		0.00 (-0.02–0.03)
	>21.5	25	33	0.10 (-0.09–0.30)		0.08 (-0.08–0.25)		0.00 (-0.03–0.04)
	Per 10-fold increase in exposure	91	143	0.07 (-0.04–0.18)		0.04 (-0.05–0.13)		0.01 (-0.01–0.02)
Bacteria (CFU/m^3^)								
	<3×103	21	23	Reference		Reference		Reference
	4×104–104	21	22	-0.01 (-0.24–0.22)		0.06 (-0.10–0.22)		-0.01 (-0.05–0.03)
	2×104–3×104	19	22	-0.09 (-0.34-0.15)		-0.02 (-0.19–0.15)		-0.02 (-0.06–0.02)
	>4×104	14	18	-0.00 (-0.28–0.27)		0.06 (-0.13–0.25)		-0.01 (-0.06–0.04)
	Per 10-fold increase in exposure	54	85	-0.03 (-0.16–0.10)		-0.04 (-0.13–0.05)		-0.00 (-0.03–0.02)
Fungi 25 °C (CFU/m^3^)								
	<5×103	19	24	Reference		Reference		Reference
	6×103–104	18	22	-0.02 (-0.24–0.21)		-0.06 (-0.21–0.10)		0.00 (-0.04–0.04)
	2×104–9×104	17	19	0.12 (-0.10–0.35)		0.09 (-0.06–0.24)		0.01 (-0.03–0.05)
	>105	14	20	0.02 (-0.24–0.27)		0.17 (-0.01–0.35)		-0.03 (-0.07–0.02)
	Per 10-fold increase in exposure	54	85	0.01 (-0.08–0.09)		0.05 (-0.01–0.11)		-0.01 (-0.02–0.01)
Fungi 37 °C (CFU/m^3^)								
	<2×102	23	23	Reference		Reference		Reference
	3×102–8×102	17	21	0.05 (-0.18–0.27)		0.07 (-0.08–0.22)		0.00 (-0.04–0.04)
	9×102–2×103	20	21	0.05 (-0.18–0.28)		0.17 (0.02–0.31)		-0.01 (-0.05–0.03)
	>3×103	15	20	-0.01 (-0.26–0.24)		0.01 (-0.15–0.18)		-0.01 (-0.05–0.03)
	Per 10-fold increase in exposure	54	85	-0.02 (-0.14–0.09)		0.02 (-0.07–0.10)		-0.01 (-0.03–0.01)

^a^ Linear mixed effect model with time of measurement, season, smoking, allergy and asthma status, body mass index, and age as fixed effects and worker as random effect. Change measured as afternoon measurement minus morning measurement. β-coefficients of the categorical exposure models express the absolute difference compared with the lowest quartile, and the β-coefficients of the continuous exposure models express the difference per 10-fold increase in exposure.

### Associations between bioaerosol exposure levels and inflammatory markers

**Figure 2:**
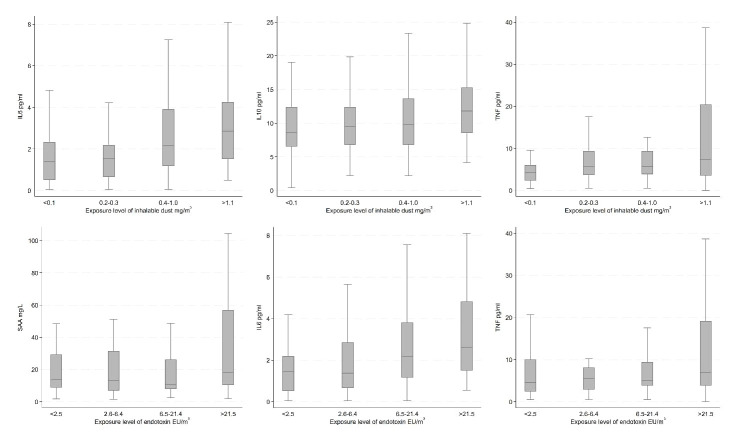
Unadjusted inflammatory marker concentrations by exposure level of inhalable dust and endotoxin among recycling workers.

**Figure 3:**
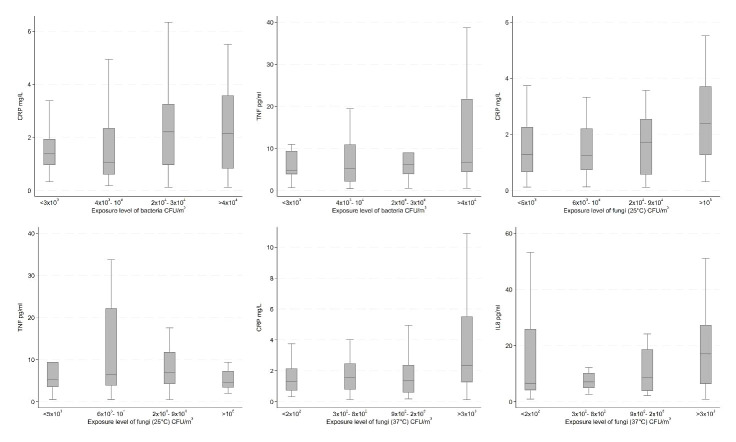
Unadjusted inflammatory marker concentrations by exposure level of bacteria, fungi (25 °C), and fungi (37 °C) among recycling workers.

In the unadjusted analyses, we observed increasing concentrations of CRP by levels of bacteria and fungi (25 and 37 °C); SAA by endotoxin; IL6 by inhalable dust and endotoxin; IL8 by fungi (37 °C); IL10 by inhalable dust; and TNF by inhalable dust, endotoxin, and bacteria; all 12 results being depicted in figures 2 and 3. On the other hand, we observed decreasing concentrations of TNF by levels of fungi (25 °C). Results for the remaining unadjusted analyses indicating no association between bioaerosol exposure levels and inflammatory markers can be found in supplementary figures S6-10.

Confounder adjusted analyses showed similar results as the unadjusted, except for CRP and bacteria, SAA, IL6, and endotoxin, and TNF, endotoxin, bacteria, and fungi (25 °C) (table 3). Furthermore, increasing exposure to inhalable dust was associated with increasing IL2 concentrations. Similarly, increasing exposure to bacteria was associated with increasing SAA and IL6 concentrations. For fungi (37 °C), increasing exposure was associated with increase in SAA and TNF concentrations. However, only the association between fungi (37 °C) and TNF was statistically significant. No associations were indicated for the remaining analyses.

**Table 3:** Adjusted ratios of inflammatory marker concentrations by bioaerosols exposure levels among recycling workers.^a^

Bioaerosols		Participants	Samples	CRP (mg/L)		SAA (mg/L)		CC16 (ng/ml)		TNF (pg/ml)
				β (95% CI)		β (95% CI)		β (95% CI)		β (95% CI)
Inhalable dust (mg/m^3^)										
	<0.1	31	43 ^b^	Reference		Reference		Reference		Reference
	0.2–0.3	38	42	0.75 (0.49–1.14)		0.92 (0.56–1.51)		0.98 (0.89–1.09)		1.14 (0.68–1.90)
	0.4–1.0	37	43	0.77 (0.49–1.21)		0.81 (0.48–1.37)		0.96 (0.86–1.08)		1.38 (0.80–2.37)
	>1.1	32	42	0.82 (0.46–1.47)		1.02 (0.55–1.87)		0.96 (0.83–1.11)		1.63 (0.89–2.96)
	Per 10-fold increase in exposure	101	170 ^b^	0.90 (0.63–1.28)		1.11 (0.77–1.61)		0.99 (0.90–1.08)		1.23 (0.85–1.77)
Endotoxin (EU/m^3^)										
	<2.5	31	43 ^b^	Reference		Reference		Reference		Reference
	2.6–6.4	38	42	**0.55 (0.38–0.80)**		0.68 (0.43–1.09)		0.95 (0.86–1.04)		1.07 (0.65–1.74)
	6.5–21.4	37	43	**0.50 (0.32–0.79)**		0.55 (0.32–0.94)		0.94 (0.84–1.06)		0.77 (0.44–1.35)
	>21.5	31	42	**0.54 (0.31–0.94)**		1.23 (0.67–2.23)		1.01 (0.87–1.16)		0.97 (0.51–1.83)
	Per 10-fold increase in exposure	101	170 ^b^	0.90 (0.66–1.22)		1.18 (0.84–1.65)		1.04 (0.96–1.12)		1.11 (0.79–1.55)
Bacteria (CFU/m^3^)										
	<3×103	23	26 ^b^	Reference		Reference		Reference		Reference
	4×104–104	23	25	1.37 (0.90–2.07)		1.70 (0.92–3.16)		0.93 (0.84–1.03)		0.83 (0.43–1.61)
	2×104–3×104	22	25	1.04 (0.68–1.57)		1.07 (0.57–2.02)		1.01 (0.91–1.12)		0.69 (0.33–1.44)
	>4×104	19	25	1.00 (0.57–1.76)		**2.22 (1.02–4.85)**		1.01 (0.87–1.16)		0.89 (0.36–2.21)
	Per 10-fold increase in exposure	59	101 ^b^	0.90 (0.68–1.20)		1.38 (0.94–2.01)		1.05 (0.97–1.12)		1.08 (0.66–1.78)
Fungi 25 °C (CFU/m^3^)										
	<5×103	19	26 ^b^	Reference		Reference		Reference		Reference
	6×103–104	20	25	1.02 (0.58–1.79)		1.41 (0.69–2.92)		0.99 (0.87–1.13)		0.90 (0.44–1.84)
	2×104–9×104	21	25	1.25 (0.76–2.06)		0.98 (0.50–1.93)		0.96 (0.85–1.08)		1.54 (0.81–2.93)
	>105	18	25	1.52 (0.76–3.04)		1.27 (0.55–2.94)		0.86 (0.72–1.02)		0.72 (0.32–1.62)
	Per 10-fold increase in exposure	59	101 ^b^	1.21 (0.96–1.52)		1.02 (0.77–1.35)		0.99 (0.93–1.05)		0.95 (0.73–1.24)
Fungi 37 °C (CFU/m^3^)										
	<2×102	24	26 ^b^	Reference		Reference		Reference		Reference
	3×102–8×102	21	25	**1.70 (1.13–2.56)**		1.63 (0.89–3.00)		0.96 (0.87–1.06)		1.11 (0.60–2.05)
	9×102–2×103	23	25	1.43 (0.97–2.10)		1.61 (0.90–2.88)		0.98 (0.89–1.07)		1.35 (0.75–2.41)
	>3×103	17	25	1.67 (0.90–3.10)		1.43 (0.67–3.05)		1.09 (0.94–1.25)		**2.48 (1.19–5.15)**
	Per 10-fold increase in exposure	59	101 ^b^	1.27 (0.97–1.65)		1.22 (0.87–1.72)		1.01 (0.95–1.08)		**1.40 (1.01–1.96)**

Season did not modify the effect of the five exposures. The different bioaerosol components were moderately correlated (supplementary table S1).

**Table 3:** Continued.

Bioaerosols		IL1B (pg/ml)		IL2 (pg/ml)		IL6 (pg/ml)		IL8 (pg/ml)		IL10 (pg/ml)
		β (95% CI)		β (95% CI)		β (95% CI)		β (95% CI)		β (95% CI)
Inhalable dust (mg/m^3^)										
	<0.1	Reference		Reference		Reference		Reference		Reference
	0.2–0.3	**0.64 (0.41–0.99)**		1.03 (0.50–2.14)		1.06 (0.62–1.81)		0.75 (0.48–1.15)		1.02 (0.82–1.26)
	0.4–1.0	0.83 (0.53–1.30)		1.97 (0.94–4.12)		1.36 (0.78–2.37)		0.98 (0.62–1.55)		1.03 (0.83–1.28)
	>1.1	0.93 (0.57–1.52)		1.70 (0.78–3.75)		1.52 (0.84–2.76)		1.08 (0.65–1.79)		1.16 (0.91–1.48)
	Per 10-fold increase in exposure	0.97 (0.72–1.30)		1.40 (0.87–2.25)		1.15 (0.81–1.65)		1.02 (0.75–1.40)		1.09 (0.95–1.27)
Endotoxin (EU/m^3^)										
	<2.5	Reference		Reference		Reference		Reference		Reference
	2.6–6.4	0.80 (0.52–1.24)		1.17 (0.60–2.28)		0.88 (0.52–1.50)		1.11 (0.73–1.70)		0.84 (0.68–1.03)
	6.5–21.4	0.82 (0.51–1.31)		0.67 (0.32–1.41)		1.25 (0.71–2.21)		0.80 (0.51–1.28)		0.96 (0.77–1.20)
	>21.5	0.93 (0.55–1.57)		0.80 (0.36–1.80)		1.20 (0.64–2.24)		1.09 (0.64–1.86)		0.97 (0.76–1.25)
	Per 10-fold increase in exposure	1.01 (0.76–1.34)		0.93 (0.60–1.42)		1.11 (0.79–1.56)		1.02 (0.77–1.37)		1.05 (0.92–1.20)
Bacteria (CFU/m^3^)										
	<3×103	Reference		Reference		Reference		Reference		Reference
	4×104–104	1.00 (0.58–1.70)		1.94 (0.76–4.95)		1.64 (0.89–3.04)		0.96 (0.60–1.55)		1.08 (0.82–1.42)
	2×104–3×104	1.00 (0.58–1.73)		1.40 (0.52–3.74)		1.88 (0.99–3.60)		0.70 (0.42–1.15)		1.12 (0.84–1.49)
	>4×104	0.86 (0.45–1.61)		1.57 (0.55–4.48)		1.53 (0.76–3.11)		0.83 (0.45–1.54)		0.98 (0.71–1.36)
	Per 10-fold increase in exposure	1.02 (0.75–1.38)		1.18 (0.73–1.92)		1.27 (0.90–1.78)		0.99 (0.72–1.37)		1.04 (0.89–1.22)
Fungi 25 °C (CFU/m^3^)										
	<5×103	Reference		Reference		Reference		Reference		Reference
	6×103–104	1.11 (0.63–1.97)		1.30 (0.54–3.10)		1.15 (0.61–2.17)		1.55 (0.90–2.66)		1.04 (0.77–1.40)
	2×104–9×104	1.75 (1.05–2.94)		1.62 (0.73–3.59)		1.46 (0.80–2.65)		1.29 (0.78–2.12)		1.19 (0.90–1.56)
	>105	1.18 (0.62–2.25)		0.62 (0.23–1.66)		0.93 (0.46–1.89)		0.87 (0.47–1.62)		1.03 (0.75–1.43)
	Per 10-fold increase in exposure	1.01 (0.82–1.25)		0.92 (0.66–1.28)		1.04 (0.83–1.31)		0.92 (0.75–1.13)		0.98 (0.88–1.09)
Fungi 37 °C (CFU/m^3^)										
	<2×102	Reference		Reference		Reference		Reference		Reference
	3×102–8×102	1.33 (0.79–2.23)		1.23 (0.52–2.88)		1.05 (0.57–1.94)		1.22 (0.77–1.92)		1.10 (0.84–1.43)
	9×102–2×103	1.14 (0.70–1.84)		0.96 (0.43–2.15)		0.86 (0.48–1.54)		1.20 (0.78–1.84)		0.91 (0.70–1.18)
	>3×103	0.76 (0.41–1.41)		0.81 (0.31–2.07)		1.05 (0.52–2.13)		**2.05 (1.18–3.56)**		0.94 (0.69–1.28)
	Per 10-fold increase in exposure	1.01 (0.76–1.35)		0.93 (0.61–1.43)		1.02 (0.74–1.39)		1.28 (0.99–1.64)		0.99 (0.86–1.14)

^a^ Linear mixed effect model with time of measurement, season, smoking, allergy and asthma status, BMI, and age as fixed effects and worker as random effect. Presented as adjusted β-coefficients (Exp(β)) with 95% confidence interval (CI). Exp(β)-coefficients of the categorical exposure models express the ratio between the means compared with the lowest quartile, and continuously the exp(β)-coefficients of the continuous exposure models express the ratio between the means per 10-fold increase in exposure. Statistically significant results marked with bold. ^b^ For one sample there was not enough serum to measure IL1-β, IL-2, IL-6, IL-8, IL-10, and TNF-α.

### Associations between production characteristics, work-shift change in lung function and inflammatory markers

The absolute work-shift change in FEV1, FVC, and FEV1/FVC were -0.06 L/min, -0.09 L/min, and 0.00 respectively among production workers, and -0.06 L/min, -0.05 L/min, and 0.00 L/min among administrative workers (supplementary table S2). In the adjusted analysis, no clear associations between production characteristics and lung function were observed.

Overall, there was a slightly higher (1.13–1.82-fold) concentrations of SAA, IL1B, IL2, IL6, IL10, and TNF among production workers compared with administrative workers, although only statistically significant for IL10 (supplementary table S3). However, CRP and CC16 concentrations were slightly lower (0.75 and 0.94) among production workers compared with administrative workers. The proportion of individual measurements below MDD varied across inflammatory markers with the smallest proportion observed for CRP, SAA, CC16, and IL8 (0-5%) and highest for IL2 (57% for production and 76% for administrative workers)

## Discussion

This study of recycling works did not indicate any acute effect on lung function (FEV1, FVC, and FEV1/FVC) of bioaerosol exposure. Increasing concentrations of IL2, IL6, IL10, and TNF were associated with increasing levels of inhalable dust, SAA and IL6 with bacteria, CRP with fungi (25 and 37 °C), and SAA, IL8, and TNF with fungi (37 °C). However, only the association between fungi (37 °C) and TNF was statistically significant.

### Comparison with previous studies

Exposure levels of inhalable dust, endotoxin, bacteria, or fungi in our study were in the range of those found within the waste, wood, sewage, greenhouse, and bioenergy industry, and below levels experienced among farmers and compost workers (7, 9, 10, 14, 15, 21, 28–33).

Previous studies of recycling workers and bioaerosol-exposed workers in other industries have provided contradicting evidence of an association between dust and endotoxin levels and acute lung function impairment (7, 9, 10, 28, 30–32). Among studies with higher exposure levels of endotoxin than we observed, Eduard et al (30), observed among farmers a statistically significantly decline in FEV1 at average exposure levels of 31×10^3^ EU/m^3^. Bunger et al (7), observed among compost workers statistically significant decrease in FVC after five years of follow-up at average levels of 16 ng/m^3^. Thus, the lack of association we observed may be due to the low concentrations of endotoxins to which the workers were exposed. An association between endotoxin exposure below 100 EU/m^3^ and respiratory symptoms has been suggested (34). Limited evidence exists of accelerated long-term lung function decline following organic dust exposure (35).

IL8 concentrations in nasal lavage fluid and sputum have previously, among waste workers, been associated with levels of total dust and endotoxins (14, 28). We could not replicate this for serum IL8. IL6 and TNF concentrations measured in nasal lavage fluid have previously shown no association with total and inhalable dust and endotoxin among waste and woodworkers (14, 33). We observed that IL6 and TNF concentrations tended to increase with increasing inhalable dust levels. Serum concentrations of CRP, SAA, and CC16 have shown contradicting associations with levels of dust, endotoxin, bacteria, and fungi among workers recycling biowaste and greenhouse workers (15, 29). Differences across studies may be explained by differences in dust composition, but also unexplained random variation.

The limited and contradicting evidence of associations between fungi, bacteria, lung function, TNF, IL6, and IL8 in our and previous studies is reflected in conclusions from two recent reviews, aiming unsuccessfully at deriving health-based occupational exposure limits (OELs) for fungi and bacteria based on human and animal experimental evidence (36, 37).

### Strengths and limitations

Company and worker participation was voluntary. However, none of the companies monitored regularly bioaerosol levels or lung function and inflammatory markers of the workers, and selective company or worker participation that may have biased findings is unlikely.

The full-shift quantitative exposure data and the quantitative outcome data allowed the assessment of exposure-response relations, a core criterion for assessing causality (38). The quality of lung function analyses adhered to ATS/ERS criteria (19, 20) and was also an important strength.

We investigated the work-shift change in lung function and were thus able to investigate the acute effect of exposure on lung function. We used the cross-shift study design in order to use individuals as their own control when exploring the acute effect of bioaerosol exposure on lung function. Due to the diurnal variation, we may overlook effects, as the diurnal lung function pattern means a higher lung function in the afternoon compared to the morning (39).

On the other hand, we only had one blood sample per worker and were not able to assess short-term changes in the inflammatory markers. However, all analyzed inflammatory markers have short plasma half-life (<4 hours), except for CRP (40–46). We therefore assume our results reflect acute effects, even if we were not able to address whether these were transient or persistent.

The many inflammatory markers and exposures analyzed opened the possibility of detecting exposure-related markers until now unrecognized. However, it also increased the risk of false positive findings. We were not able to adjust for multiple testing as estimates, confidence intervals, and P-values are not independent within or between analyses.

The different bioaerosol components of this study were moderately correlated. Findings for one component may therefore be, at least partly, explained by one or more of the other components. However, we abstained from mutual adjustments to avoid over-adjustment. We adjusted for well-documented predictors of lung function and inflammatory marker concentrations (25–27) and confounding can hardly explain our findings, even if residual confounding cannot be ruled out. The participants were at work during the measurement day, and we, therefore, assume they are free of diseases that may have affected our results. Different microbial species composition may affect inflammation differently, and furthermore varies across seasons (8). However, we observed no effect modification of season.

### Concluding remarks

This study of recycling workers exposed to inhalable dust, endotoxin, bacteria, or fungi at levels generally below levels experienced among farmers and compost workers and above background levels did not indicate any acute effect on lung function. Several serum inflammatory markers tended to increase with increasing exposure, which may represent a systemic effect. Future research should combine data from bioaerosol-exposed workers within and outside the recycling industry, including the current, to uncover health risks that may form the basis for health-based occupational exposure limits.

## Supplementary material

Supplementary material

## Data Availability

The data underlying this paper cannot be shared publicly due to the privacy of the individuals and companies who participated in the study, but data can be shared in an anonymized form on reasonable request to the corresponding author.

## References

[1] Eurostat. Municipal waste statistics. 2023. Available from: https://ec.europa.eu/eurostat/statistics-explained/index.php?title=Municipal_waste_statistics#Municipal_waste_generation

[2] European Commission. A European Green Deal. 2019. Available from: https://commission.europa.eu/strategy-and-policy/priorities-2019-2024/european-green-deal_en

[3] Statistics Denmark. Employeed in the waste and recycling industry from 2008-2022 (in Danish). 2022. Available from: https://www.statistikbanken.dk/RAS309

[4] Douwes J, Eduard W, Thorne PS. Bioaerosols. In: Heggenhougen HK, editor. International Encyclopedia of Public Health. Oxford: Academic Press; 2008. p. 287-97.

[5] Liebers V, Raulf-Heimsoth M, Brüning T. Health effects due to endotoxin inhalation (review) [review] [review]. Arch Toxicol 2008 Apr;82(4):203–10. 10.1007/s00204-008-0290-1 18322674

[6] Athanasiou M, Makrynos G, Dounias G. Respiratory health of municipal solid waste workers. Occup Med (Lond) 2010 Dec;60(8):618–23. 10.1093/occmed/kqq127 20819804

[7] Bünger J, Schappler-Scheele B, Hilgers R, Hallier E. A 5-year follow-up study on respiratory disorders and lung function in workers exposed to organic dust from composting plants. Int Arch Occup Environ Health 2007 Feb;80(4):306–12. 10.1007/s00420-006-0135-2 16897096

[8] Madsen AM, Raulf M, Duquenne P, Graff P, Cyprowski M, Beswick A et al. Review of biological risks associated with the collection of municipal wastes. Sci Total Environ 2021 Oct;791:148287. 10.1016/j.scitotenv.2021.148287 34139489

[9] Sigsgaard T, Abel A, Donbaek L, Malmros P. Lung function changes among recycling workers exposed to organic dust. Am J Ind Med 1994 Jan;25(1):69–72. 10.1002/ajim.4700250118 8116657

[10] Rylander R, Thorn J, Attefors R. Airways inflammation among workers in a paper industry. Eur Respir J 1999 May;13(5):1151–7. 10.1034/j.1399-3003.1999.13e35.x 10414419

[11] Sigsgaard T, Jensen LD, Abell A, Würtz H, Thomsen G. Endotoxins isolated from the air of a Danish paper mill and the relation to change in lung function: an 11-year follow-up. Am J Ind Med 2004 Oct;46(4):327–32. 10.1002/ajim.20068 15376218

[12] Timm M, Madsen AM, Hansen JV, Moesby L, Hansen EW. Assessment of the total inflammatory potential of bioaerosols by using a granulocyte assay. Appl Environ Microbiol 2009 Dec;75(24):7655–62. 10.1128/AEM.00928-09 19837831 PMC2794106

[13] Poole CJ, Basu S. Systematic Review: occupational illness in the waste and recycling sector. Occup Med (Lond) 2017 Dec;67(8):626–36. 10.1093/occmed/kqx153 29165683 PMC5927023

[14] Wouters IM, Hilhorst SK, Kleppe P, Doekes G, Douwes J, Peretz C et al. Upper airway inflammation and respiratory symptoms in domestic waste collectors. Occup Environ Med 2002 Feb;59(2):106–12. 10.1136/oem.59.2.106 11850553 PMC1740259

[15] Rasmussen PU, Frederiksen MW, Carøe TK, Madsen AM. Health symptoms, inflammation, and bioaerosol exposure in workers at biowaste pretreatment plants. Waste Manag 2023 Jul;167:173–82. 10.1016/j.wasman.2023.05.042 37269581

[16] Legal Information. Executive Order on Waste (in Danish). 2021. Available from: https://www.retsinformation.dk/eli/lta/2021/2512

[17] Ministry of Environment of Denmark. Action Plan for Circular Economy. 2021.

[18] Hansen KK, Schlünssen V, Broberg K, Østergaard K, Frederiksen MW, Madsen AM et al. Exposure levels of dust, endotoxin, and microorganisms in the Danish recycling industry. Ann Work Expo Health 2023 Aug;67(7):816–30. 10.1093/annweh/wxad025 37191914 PMC10410489

[19] Stanojevic S, Kaminsky DA, Miller MR, Thompson B, Aliverti A, Barjaktarevic I et al. ERS/ATS technical standard on interpretive strategies for routine lung function tests. Eur Respir J 2022 Jul;60(1):2101499. 10.1183/13993003.01499-2021 34949706

[20] Hansen MR, Jørs E, Sandbæk A, Sekabojja D, Ssempebwa JC, Mubeezi R et al. Organophosphate and carbamate insecticide exposure is related to lung function change among smallholder farmers: a prospective study. Thorax 2021 Jan;76(8):780–9. 10.1136/thoraxjnl-2020-214609 33479045 PMC8311090

[21] Basinas I, Sigsgaard T, Heederik D, Takai H, Omland Ø, Andersen NT et al. Exposure to inhalable dust and endotoxin among Danish livestock farmers: results from the SUS cohort study. J Environ Monit 2012 Feb;14(2):604–14. 10.1039/C1EM10576K 22159073

[22] Whitcomb BW, Schisterman EF. Assays with lower detection limits: implications for epidemiological investigations. Paediatr Perinat Epidemiol 2008 Nov;22(6):597–602. 10.1111/j.1365-3016.2008.00969.x 19000298 PMC2723785

[23] StataCorp. Stata Statistical Software: Release 17. College Station, TX: StataCorp LLC; 2021.

[24] Hughes JP. Mixed effects models with censored data with application to HIV RNA levels. Biometrics 1999 Jun;55(2):625–9. 10.1111/j.0006-341X.1999.00625.x 11318225

[25] Rudnicka AR, Rumley A, Lowe GD, Strachan DP. Diurnal, seasonal, and blood-processing patterns in levels of circulating fibrinogen, fibrin D-dimer, C-reactive protein, tissue plasminogen activator, and von Willebrand factor in a 45-year-old population. Circulation 2007 Feb;115(8):996–1003. 10.1161/CIRCULATIONAHA.106.635169 17296859

[26] Ellulu MS, Patimah I, Khaza’ai H, Rahmat A, Abed Y. Obesity and inflammation: the linking mechanism and the complications. Arch Med Sci 2017 Jun;13(4):851–63. 10.5114/aoms.2016.58928 28721154 PMC5507106

[27] Bui DS, Lodge CJ, Burgess JA, Lowe AJ, Perret J, Bui MQ et al. Childhood predictors of lung function trajectories and future COPD risk: a prospective cohort study from the first to the sixth decade of life. Lancet Respir Med 2018 Jul;6(7):535–44. 10.1016/S2213-2600(18)30100-0 29628376

[28] Heldal KK, Halstensen AS, Thorn J, Eduard W, Halstensen TS. Airway inflammation in waste handlers exposed to bioaerosols assessed by induced sputum. Eur Respir J 2003 Apr;21(4):641–5. 10.1183/09031936.03.00059702 12762350

[29] Madsen AM, Thilsing T, Bælum J, Garde AH, Vogel U. Occupational exposure levels of bioaerosol components are associated with serum levels of the acute phase protein Serum Amyloid A in greenhouse workers. Environ Health 2016 Jan;15:9. 10.1186/s12940-016-0090-7 26792395 PMC4719338

[30] Eduard W, Pearce N, Douwes J. Chronic bronchitis, COPD, and lung function in farmers: the role of biological agents. Chest 2009 Sep;136(3):716–25. 10.1378/chest.08-2192 19318669

[31] Heldal KK, Madsø L, Huser PO, Eduard W. Exposure, symptoms and airway inflammation among sewage workers. Ann Agric Environ Med 2010;17(2):263–8. 21186769

[32] Schlünssen V, Madsen AM, Skov S, Sigsgaard T. Does the use of biofuels affect respiratory health among male Danish energy plant workers? Occup Environ Med 2011 Jul;68(7):467–73. 10.1136/oem.2009.054403 21098831

[33] Roponen M, Seuri M, Nevalainen A, Hirvonen MR. Fungal spores as such do not cause nasal inflammation in mold exposure. Inhal Toxicol 2002 May;14(5):541–9. 10.1080/089583701753678616 12028807

[34] Farokhi A, Heederik D, Smit LA. Respiratory health effects of exposure to low levels of airborne endotoxin - a systematic review. Environ Health 2018 Feb;17(1):14. 10.1186/s12940-018-0360-7 29422043 PMC5806377

[35] Bolund AC, Miller MR, Sigsgaard T, Schlünssen V. The effect of organic dust exposure on long-term change in lung function: a systematic review and meta-analysis. Occup Environ Med 2017 Jul;74(7):531–42. 10.1136/oemed-2016-103963 28404791

[36] Walser SM, Gerstner DG, Brenner B, Bünger J, Eikmann T, Janssen B et al. Evaluation of exposure-response relationships for health effects of microbial bioaerosols - A systematic review. Int J Hyg Environ Health 2015 Oct;218(7):577–89. 10.1016/j.ijheh.2015.07.004 26272513

[37] Zamfir M, Gerstner DG, Walser SM, Bünger J, Eikmann T, Heinze S et al. A systematic review of experimental animal studies on microbial bioaerosols: dose-response data for the derivation of exposure limits. Int J Hyg Environ Health 2019 Mar;222(2):249–59. 10.1016/j.ijheh.2018.11.004 30497988

[38] Hill AB. The Environment and Disease: association or Causation? Proc R Soc Med 1965 May;58(5):295–300. 10.1177/003591576505800503 14283879 PMC1898525

[39] Borsboom GJ, van Pelt W, van Houwelingen HC, van Vianen BG, Schouten JP, Quanjer PH. Diurnal variation in lung function in subgroups from two Dutch populations: consequences for longitudinal analysis. Am J Respir Crit Care Med 1999 Apr;159(4 Pt 1):1163–71. 10.1164/ajrccm.159.4.9703106 10194161

[40] Pepys MB, Hirschfield GM. C-reactive protein: a critical update. J Clin Invest 2003 Jun;111(12):1805–12. 10.1172/JCI200318921 12813013 PMC161431

[41] Tape C, Kisilevsky R. Apolipoprotein A-I and apolipoprotein SAA half-lives during acute inflammation and amyloidogenesis. Biochim Biophys Acta 1990 Apr;1043(3):295–300. 10.1016/0005-2760(90)90030-2 2108727

[42] Broeckaert F, Clippe A, Knoops B, Hermans C, Bernard A. Clara cell secretory protein (CC16): features as a peripheral lung biomarker. Ann N Y Acad Sci 2000;923:68–77. 10.1111/j.1749-6632.2000.tb05520.x 11193780

[43] Donohue JH, Rosenberg SA. The fate of interleukin-2 after in vivo administration. J Immunol 1983 May;130(5):2203–8. 10.4049/jimmunol.130.5.2203 6601147

[44] Waage A, Brandtzaeg P, Halstensen A, Kierulf P, Espevik T. The complex pattern of cytokines in serum from patients with meningococcal septic shock. Association between interleukin 6, interleukin 1, and fatal outcome. J Exp Med 1989 Jan;169(1):333–8. 10.1084/jem.169.1.333 2783334 PMC2189201

[45] Huhn RD, Radwanski E, Gallo J, Affrime MB, Sabo R, Gonyo G et al. Pharmacodynamics of subcutaneous recombinant human interleukin-10 in healthy volunteers. Clin Pharmacol Ther 1997 Aug;62(2):171–80. 10.1016/S0009-9236(97)90065-5 9284853

[46] Liu C, Chu D, Kalantar-Zadeh K, George J, Young HA, Liu G. Cytokines: From Clinical Significance to Quantification. Adv Sci (Weinh) 2021 Aug;8(15):e2004433. 10.1002/advs.202004433 34114369 PMC8336501

