# Supplementary material for: Associations between bioaerosols, lung function work-shift changes and inflammatory markers: A study of recycling workers
Source: Scand J Work Environ Health. 2024 Nov 28;50(8):602–12. doi: 10.5271/sjweh.4187 (PMC11618316; doi:10.5271/sjweh.4187)
Supplement: Supplementary material [file SJWEH-50-602-S001.pdf]

Associations between bioaerosols, lung function work-shift changes and inflammatory markers: A study of recycling workers[1]

by Karoline Kærgaard Hansen, PhD,[2] Vivi Schlünssen, MD, Karin Broberg, PhD, Kirsten Østergaard, Lab Tech, Margit W Frederiksen, Lab Tech, Torben Sigsgaard, MD, Anne Mette Madsen, PhD, Henrik Albert Kolstad, MD

1. Supplementary material
2. Correspondence to: Karoline Kærgaard Hansen, Department of Occupational Medicine, Danish Ramazzini Centre, Aarhus University Hospital, Palle Juul-Jensens Boulevard 99, 8200 Aarhus N, Denmark or Department of Public Health, Research unit for Environment Occupation and Health, Danish Ramazzini Centre, Aarhus University, DK-8000 Aarhus C, Denmark. [E-mail: karohn@rm.dk]

Table S1 Pearson pairwise correlation coefficients for the bioaerosol exposures

Bioaerosol	Inhalable dust	Endotoxin	Bacteria	Fungi (25 °C)	Fungi (37 °C)
Inhalable dust	1				
Endotoxin	0.53	1			
Bacteria	0.52	0.46	1		
Fungi (25 °C)	-0.03	-0.01	-0.05	1	
Fungi (37 °C)	0.43	0.52	0.32	-0.02	1

Table S2 Absolute adjusted work-shift change in lung function by production characteristics among recycling workers

			Change in FEV1 (L/min)			Change in FVC (L/min)			Change in FEV1/FVC		
Production characteristics	Persons	Samples	AM	SD	β[a] (95 % CI)	AM	SD	β[a] (95 % CI)	AM	SD	β[a] (95 % CI)
Administrative worker	13	19	-0.06	0.25	Reference	-0.05	0.28	Reference	0.00	0.02	Reference
Production worker	78	124	-0.06	0.38	-0.06 (-0.27-0.14)	-0.09	0.31	-0.08 (-0.25-0.10)	0.00	0.06	0.00 (-0.04-0.03)
Work task[b]											
Sorting	40	64	-0.04	0.40	-0.03 (-0.24-0.18)	-0.08	0.33	-0.06 (-0.24-0.12)	0.00	0.07	0.00 (-0.04-0.03)
Machine operation	17	25	-0.11	0.44	-0.13 (-0.37-0.11)	-0.14	0.29	-0.11 (-0.31-0.09)	0.00	0.08	-0.01 (-0.05-0.03)
Driving	22	31	-0.04	0.31	-0.07 (-0.30-0.17)	-0.08	0.32	-0.08 (-0.28-0.12)	0.01	0.03	0.00 (-0.04-0.04)
Cleaning and maintenance	3	4	-0.18	0.13	-0.08 (-0.50-0.34)	-0.15	0.11	-0.05 (-0.40-0.31)	-0.02	0.03	-0.02 (-0.09-0.05)
Waste fraction[b]											
Paper/cardboard	13	18	-0.08	0.69	-0.06 (-0.33-0.21)	-0.09	0.39	-0.07 (-0.329-0.16)	-0.01	0.12	-0.01 (-0.06-0.03)
Metal	14	19	-0.05	0.24	-0.07 (-0.34-0.20)	-0.11	0.35	-0.09 (-0.32-0.14)	0.01	0.03	0.00 (-0.05-0.04)
Plastic	8	13	-0.18	0.52	-0.16 (-0.43-0.11)	-0.13	0.20	-0.08 (-0.30-0.15)	-0.02	0.10	-0.02 (-0.06-0.02)
Biowaste	5	7	-0.06	0.19	-0.07 (-0.39-0.25)	-0.09	0.23	-0.06 (-0.33-0.21)	0.00	0.02	-0.01 (-0.06-0.05)
Electronics and hazardous[c]	13	23	-0.12	0.31	-0.06 (-0.29-0.18)	-0.17	0.45	-0.14 (-0.34-0.06)	0.00	0.04	0.01 (-0.03-0.05)
Metal/plastic/glass	13	20	0.07	0.21	0.09 (-0.18-0.36)	0.03	0.18	0.09 (-0.13-0.33)	0.01	0.03	0.00 (-0.04-0.05)
Mixed[d]	16	24	-0.06	0.18	-0.02 (-0.27-0.23)	-0.11	0.17	-0.05 (-0.26-0.16)	0.00	0.03	0.00 (-0.04-0.04)
Location/ventilation[b,e]											
No room/vehicle ventilation (indoor)	19	23	-0.02	0.26	Reference	-0.08	0.31	Reference	0.01	0.03	Reference
Room/vehicle ventilation (indoor)	47	70	-0.09	0.46	-0.07 (-0.25-0.11)	-0.11	0.35	-0.05 (-0.20-0.10)	0.00	0.08	-0.01 (-0.04-0.02)
Outdoor	20	31	-0.02	0.26	0.04 (-0.25-0.17)	-0.05	0.20	0.00 (-0.17-0.18)	0.00	0.02	-0.01 (-0.05-0.02)
Personal protective equipment (PPE)[e]											
No PPE	12	13	-0.01	0.38	Reference	-0.13	0.35	Reference	0.02	0.04	Reference
Face mask[f]	13	16	-0.13	0.19	-0.10 (-0.36-0.17)	-0.21	0.23	-0.07 (-0.29-0.15)	0.01	0.02	-0.01 (-0.06-0.03)
Other PPE[g]	66	95	-0.05	0.40	-0.02 (-0.25-0.20)	-0.07	0.31	0.11 (-0.08-0.29)	0.00	0.07	-0.03 (-0.07-0.01)

[a] Linear mixed effect model with time of measurement, season, smoking, allergy and asthma status, BMI, and age as fixed effects and worker as random effect. Change measured as afternoon measurement minus morning measurement. Presented as adjusted β-coefficients with 95% confidence interval (CI). β-coefficients express the absolute difference compared with the reference.

[b] Primary. For repeated measurements, a person can appear in two categories

[c] Classified as hazardous waste, e.g. light bulbs, paint, and spray cans

[d] More than one fraction of waste fraction, including working at a recycling station

[e] Only among production workers

[f] Filtering face piece-2 or -3 (FFP2 or FFP3) respirators, surgical masks, or air-supplied respirator

[g] Plastic apron, gloves, visor or safety goggles

Table S3 Adjusted ratios of inflammatory marker concentrations by production characteristics among recycling workers

			CRP[a] (mg/L)		SAA[a] (mg/L)		CC16[a] (ng/ml)		IL-1β[a] (pg/ml)		IL-2[a] (pg/ml)	
Production characteristics	Persons	Samples	Exp(β)[a] (95% CI)	% below MDD	Exp(β)[a] (95% CI)	% below MDD	Exp(β)[a] (95% CI)	% below MDD	Exp(β)[a] (95% CI)	% below MDD	Exp(β)[a] (95% CI)	% below MDD
Administrative worker	14	21	Reference	0	Reference	0	Reference	0	Reference	29	Reference	76
Production worker	88	149[h]	0.75 (0.36-1.55)	1	1.15 (0.57-2.31)	1	0.94 (0.77-1.15)	0	1.36 (0.80-2.33)	27	1.82 (0.77-4.35)	57
Work task[b]												
Sorting	46	78	0.77 (0.35-1.68)	1	1.08 (0.52-2.27)	1	0.91 (0.73-1.14)	0	1.51 (0.86-2.64)	11	1.73 (0.70-4.25)	28
Machine operation	19	28	0.80 (0.34-1.90)	0	1.04 (0.45-2.38)	0	0.97 (0.76-1.23)	0	1.04 (0.55-1.99)	7	1.65 (0.60-4.59)	7
Driving	25	36[h]	0.68 (0.31-1.48)	0	1.16 (0.53-2.50)	0	0.96 (0.78-1.19)	0	1.46 (0.80-2.67)	5	2.42 (0.93-6.29)	12
Cleaning and maintenance	5	7	1.21 (0.35-4.18)	0	3.21 (0.93-11.03)	0	0.84 (0.60-1.18)	0	0.96 (0.36-2.54)	1	0.63 (0.13-3.01)	3
Waste fraction[b]												
Paper/cardboard	17	28[h]	0.58 (0.23-1.43)	0	0.92 (0.39-2.21)	1	0.82 (0.63-1.06)	0	1.73 (0.90-3.33)	4	2.69 (0.95-7.60)	5
Metal	15	21	1.01 (0.39-2.61)	0	1.77 (0.72-4.35)	0	0.96 (0.72-1.27)	0	1.03 (0.52-2.01)	5	1.47 (0.48-4.55)	8
Plastic	11	17	1.15 (0.46-2.84)	0	1.47 (0.59-3.63)	0	1.04 (0.81-1.33)	0	0.99 (0.49-2.02)	5	1.58 (0.53-4.69)	4
Biowaste	6	10	0.45 (0.13-1.53)	0	0.83 (0.27-2.58)	0	0.91 (0.63-1.32)	0	1.15 (0.51-2.60)	1	1.55 (0.37-6.43)	4
Electronics and hazardous[c]	14	27	0.68 (0.27-1.71)	0	0.78 (0.33-1.85)	0	0.84 (0.64-1.12)	0	1.42 (0.77-2.63)	3	1.52 (0.55-4.25)	10
Metal/plastic/glass	13	22	0.94 (0.34-2.58)	0	1.05 (0.41-2.70)	0	1.06 (0.79-1.43)	0	1.05 (0.53-2.07)	4	1.16 (0.34-3.98)	11
Mixed[d]	16	24	**0.36 (0.14-0.89)**	1	1.24 (0.51-2.98)	0	0.79 (0.61-1.03)	0	**2.29 (1.20-4.37)**	2	2.86 (0.97-8.46)	8
Location/ventilation[b,e]												
No room/vehicle ventilation (indoor)	22	30[h]	Reference	0	Reference	1	Reference	0	Reference	8	Reference	12
Room/vehicle ventilation (indoor)	55	86	1.30 (0.79-2.12)	0	1.42 (0.82-2.48)	0	1.05 (0.94-1.17)	0	**1.59 (1.02-2.47)**	14	1.37 (0.71-2.64)	32
Outdoor	22	33	0.68 (0.36-1.29)	1	0.99 (0.50-1.97)	0	0.93 (0.80-1.08)	0	**1.72 (1.01-2.93)**	5	1.42 (0.63-3.21)	13
Personal protective equipment (PPE)[e]												
No PPE	13	15	Reference	0	Reference	0	Reference	0	Reference	3	Reference	6
Face mask[f]	14	18	1.44 (0.72-2.85)	0	1.07 (0.47-2.44)	0	0.93 (0.80-1.09)	0	0.85 (0.43-1.70)	3	1.14 (0.42-3.11)	6
Other PPE[g]	76	116[h]	1.20 (0.71-2.05)	1	1.11 (0.57-2.15)	1	0.97 (0.86-1.09)	0	0.90 (0.52-1.58)	21	1.11 (0.50-2.47)	45

[a] Linear mixed effect model with time of measurement, season, smoking, allergy and asthma status, BMI, and age as fixed effects and worker as random effect. Presented as adjusted β-coefficients (Exp(β)) with 95% confidence interval (CI). Exp(β)-coefficients express the ratio between the means compared with the reference. Statistically significant results marked with bold.

[b] Primary. For repeated measurements, a person can appear in two categories

[c] Classified as hazardous waste, e.g. light bulbs, paint, and spray cans

[d] More than one fraction of waste fraction, including working at a recycling station

[e] Only among production workers

[f] Filtering face piece-2 or -3 (FFP2 or FFP3) respirators, surgical masks, or air-supplied respirator

[g] Plastic apron, gloves, visor or safety goggles

[h] For one sample there was not enough serum to measure IL1-β, IL-2, IL-6, IL-8, IL-10, and TNF-α,

Table S3 Adjusted ratios of inflammatory marker concentrations by production characteristics among recycling workers (continued)

Production characteristics	IL-6[a] (pg/ml) Exp(β)[a] (95% CI)	% below MDD	IL-8[a] (pg/ml) Exp(β)[a] (95% CI)	% below MDD	IL-10[a] (pg/ml) Exp(β)[a] (95% CI)	% below MDD	TNF-α[a] (pg/ml) Exp(β)[a] (95% CI)	% below MDD
Administrative worker	Reference	24	Reference	5	Reference	24	Reference	14
Production worker	1.13 (0.60-2.14)	21	1.00 (0.58-1.73)	0	**1.36 (1.06-1.75)**	11	1.41 (0.72-2.74)	13
Work task[b]								
Sorting	1.16 (0.59-2.28)	11	1.07 (0.61-1.88)	0	**1.43 (1.11-1.85)**	4	1.38 (0.67-2.83)	7
Machine operation	0.93 (0.43-2.02)	4	0.75 (0.40-1.43)	0	1.21 (0.90-1.63)	2	1.47 (0.66-3.28)	2
Driving	1.21 (0.58-2.51)	4	1.21 (0.65-2.25)	0	**1.41 (1.06-1.86)**	3	1.35 (0.63-2.89)	2
Cleaning and maintenance	1.39 (0.44-4.40)	0	0.72 (0.27-1.90)	0	1.12 (0.71-1.75)	1	1.78 (0.54-5.86)	0
Waste fraction[b]								
Paper/cardboard	1.33 (0.60-2.95)	3	**2.40 (1.32-4.36)**	0	**1.57 (1.16-2.14)**	1	**3.27 (1.50-7.13)**	1
Metal	0.86 (0.38-1.97)	4	0.46 (0.25-0.85)	0	1.35 (0.99-1.86)	2	0.89 (0.40-1.99)	2
Plastic	1.23 (0.53-2.86)	1	0.82 (0.44-1.53)	0	1.18 (0.85-1.63)	2	1.16 (0.52-2.61)	1
Biowaste	1.24 (0.45-3.37)	1	1.08 (0.51-2.29)	0	1.25 (0.85-1.84)	1	1.00 (0.37-2.71)	1
Electronics and hazardous[c]	0.73 (0.34-1.58)	5	1.32 (0.75-2.33)	0	**1.34 (1.01-1.80)**	2	1.72 (0.81-3.66)	1
Metal/plastic/glass	1.26 (0.53-2.97)	4	0.61 (0.33-1.16)	0	1.24 (0.89-1.71)	2	0.62 (0.27-1.46)	4
Mixed[d]	1.79 (0.81-3.97)	0	1.35 (0.74-2.46)	0	**1.69 (1.25-2.30)**	1	2.15 (0.98-4.74)	1
Location/ventilation[b,e]								
Room/vehicle ventilation (indoor)	Reference	5	Reference	0	Reference	4	Reference	3
No room/vehicle ventilation (indoor)	1.16 (0.67-2.00)	14	1.21 (0.80-1.85)	0	1.11 (0.90-1.37)	5	1.04 (0.62-1.74)	8
Outdoor	1.06 (0.55-2.03)	3	1.16 (0.69-1.93)	0	1.17 (0.90-1.51)	2	1.03 (0.54-1.93)	2
Personal protective equipment (PPE)[e]								
No PPE	Reference	3	Reference	0	Reference	2	Reference	2
Face mask[f]	1.10 (0.48-2.52)	2	1.36 (0.72-2.56)	0	1.24 (0.90-1.71)	1	1.60 (0.74-3.44)	1
Other PPE[g]	1.19 (0.61-2.36)	16	1.31 (0.78-2.20)	0	1.06 (0.81-1.38)	9	1.53 (0.82-2.86)	9

[a] Linear mixed effect model with hour of measurement, season, smoking, allergy and asthma status, BMI, and age as fixed effects and worker as random effect. Presented as adjusted β-coefficients (Exp(β)) with 95% confidence interval (CI). Exp(β)-coefficients express the ratio between the means compared with the reference. Statistically significant results marked with bold.

[b] Primary. For repeated measurements, a person can appear in two categories

[c] Classified as hazardous waste, e.g. light bulbs, paint, and spray cans

[d] More than one fraction of waste fraction, including working at a recycling station

[e] Only among production workers

[f] Filtering face piece-2 or -3 (FFP2 or FFP3) respirators, surgical masks, or air-supplied respirator

[g] Plastic apron, gloves, visor or safety goggles

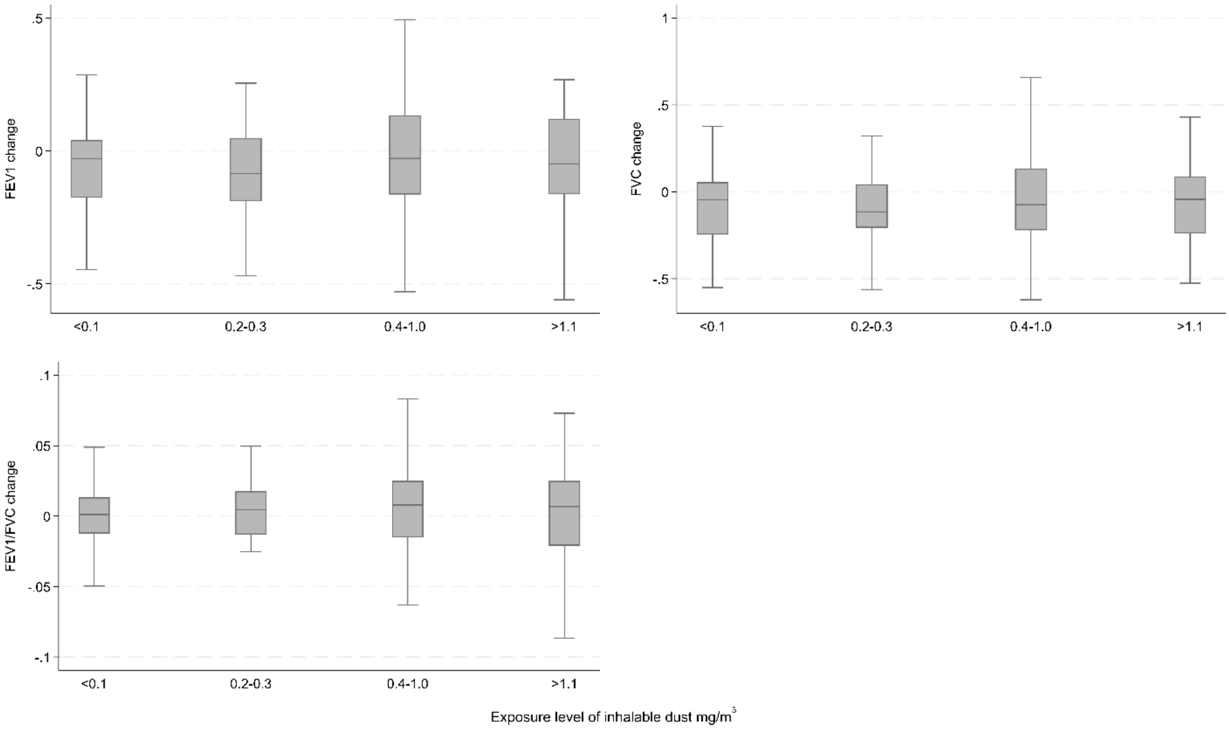

Figure S1 Unadjusted work-shift change in lung function change by exposure level of inhalable dust among recycling workers

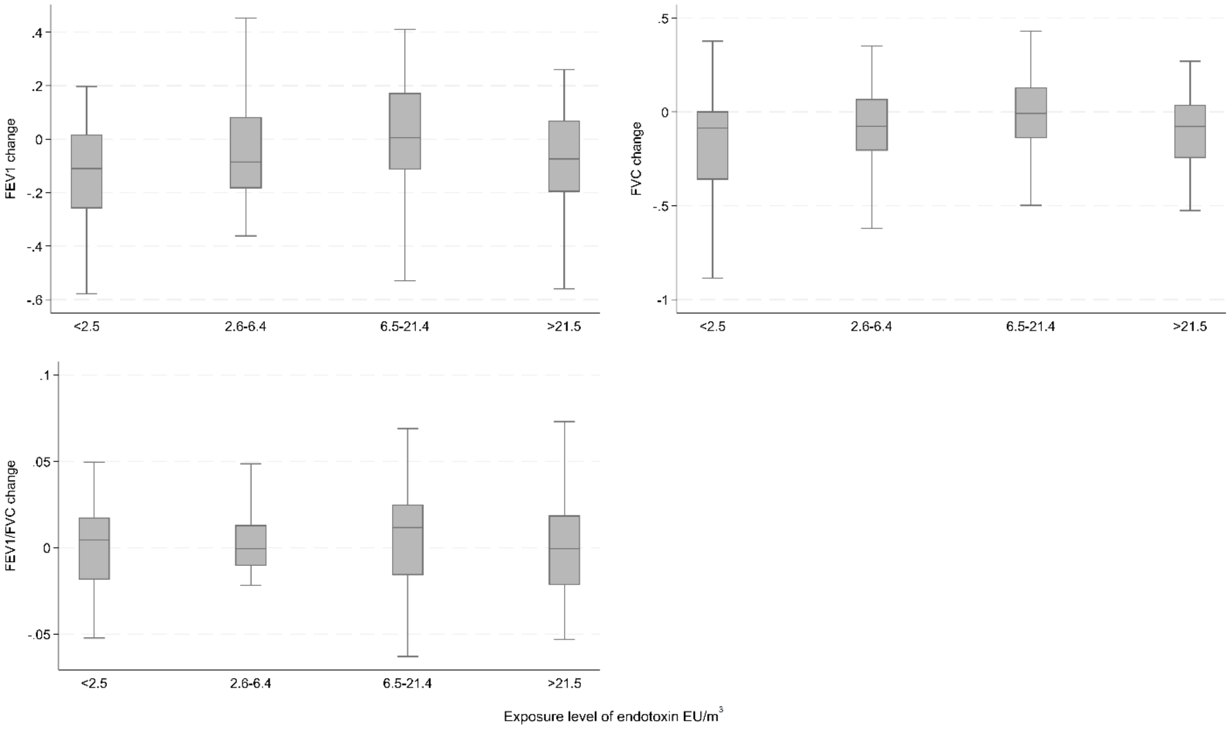

Figure S2 Unadjusted work-shift change in lung function change by exposure level of endotoxin among recycling workers

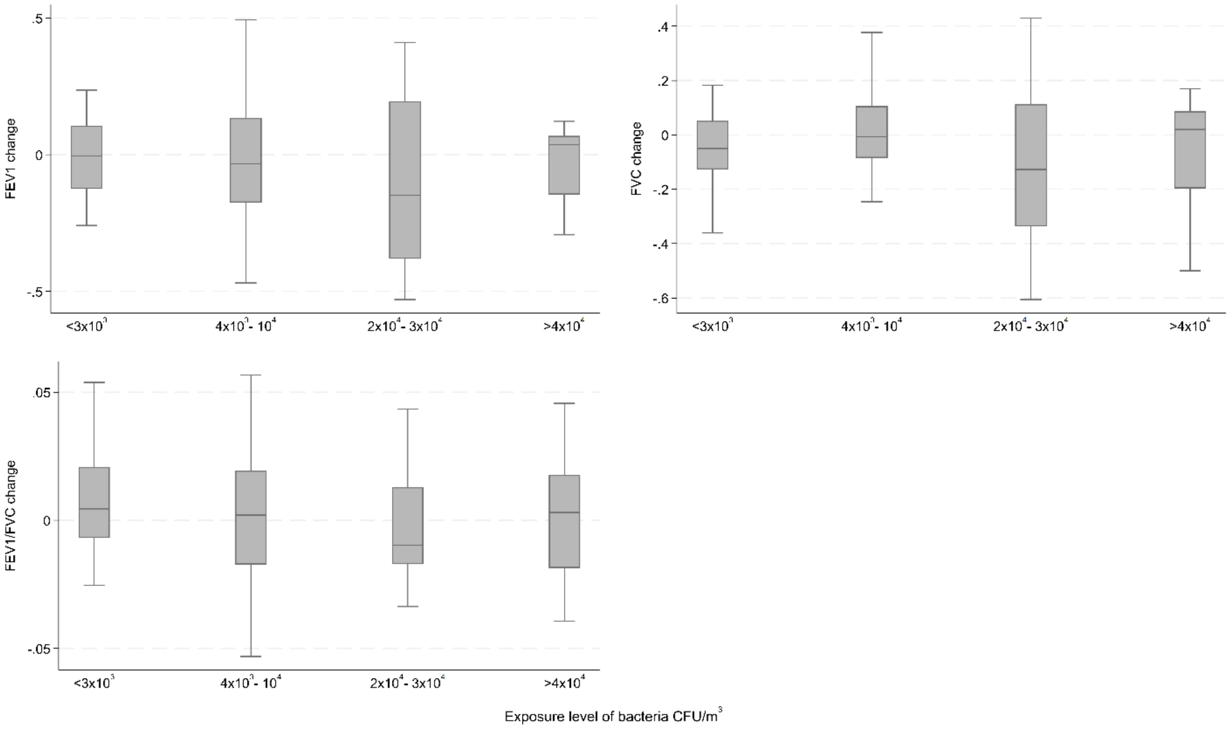

Figure S3 Unadjusted work-shift change in lung function change by exposure level of bacteria among recycling workers

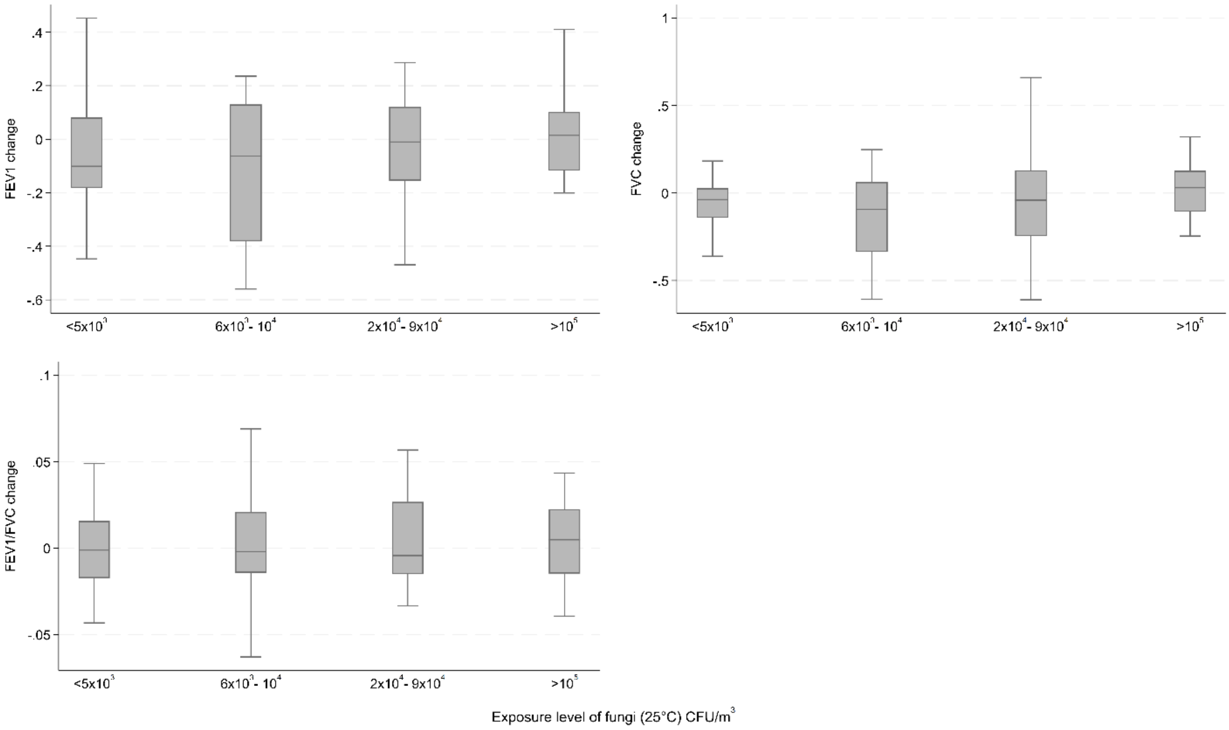

Figure S4 Unadjusted work-shift change in lung function change by exposure level of fungi (25 °C) among recycling workers

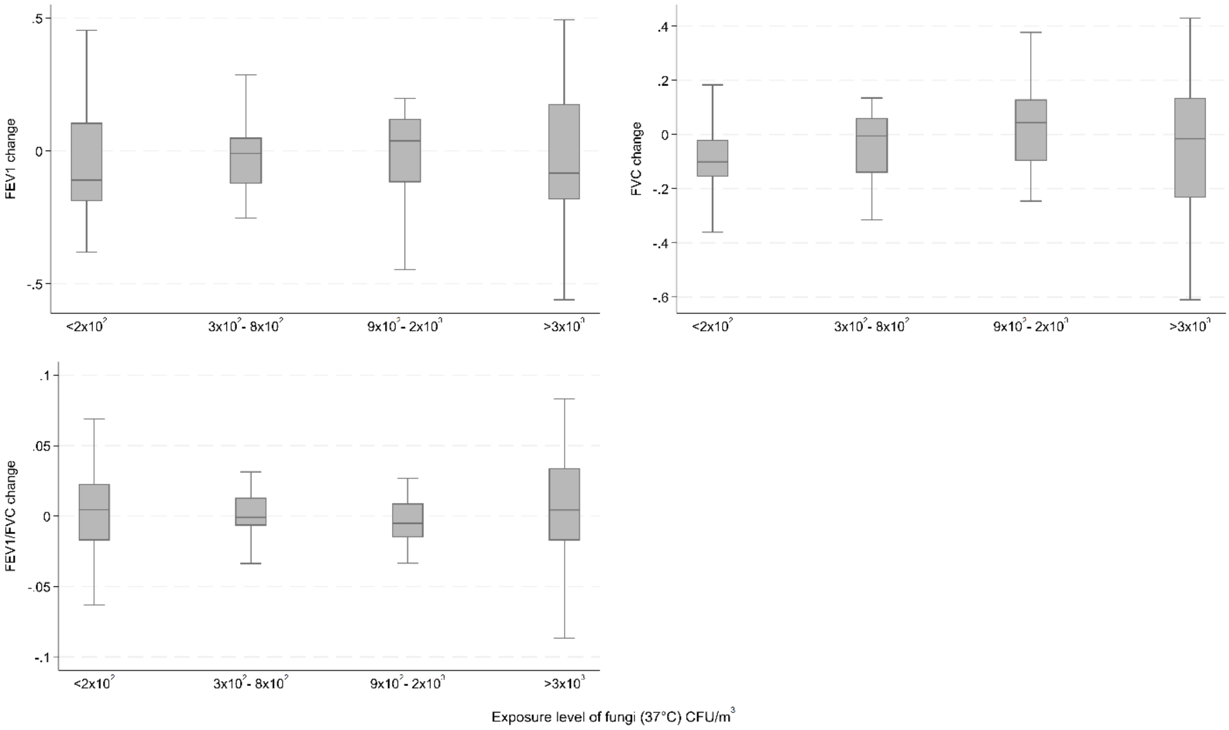

Figure S5 Unadjusted work-shift change in lung function change by exposure level of fungi (37 °C) among recycling workers

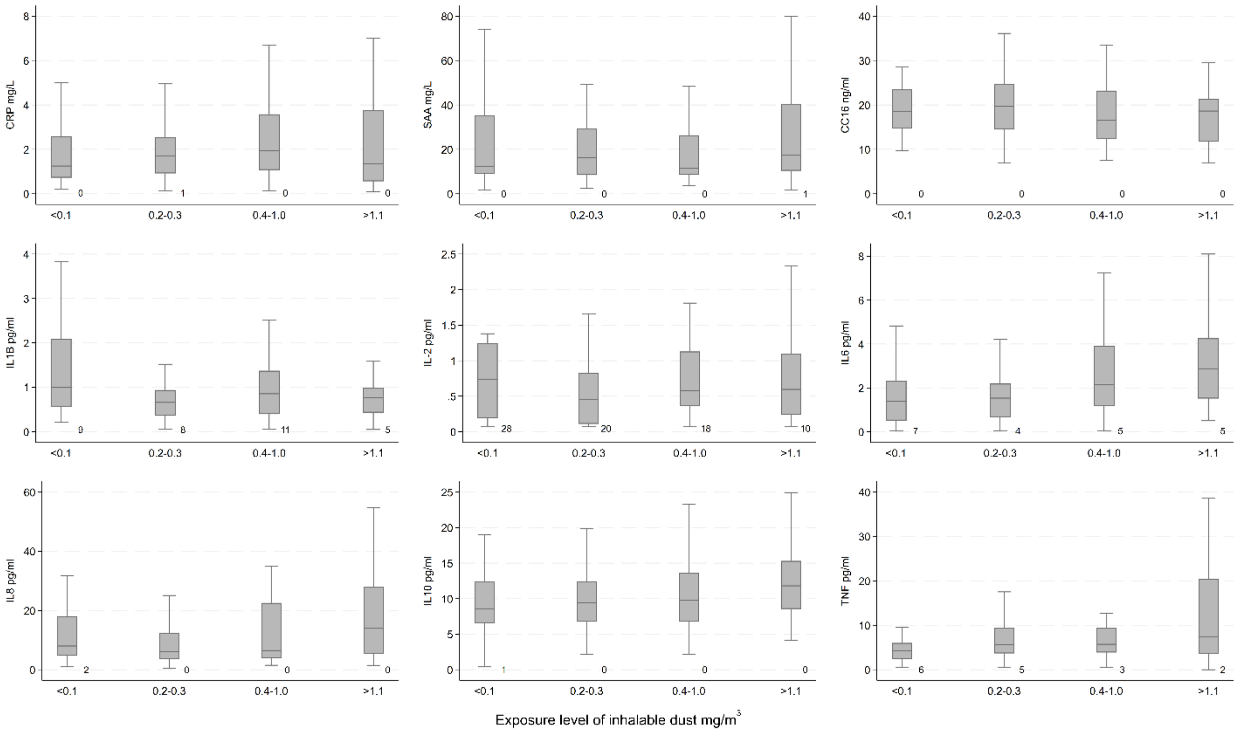

Figure S6 Unadjusted inflammatory marker concentrations by exposure level of inhalable dust among recycling workers. Number of measurements below MDD at the bottom to the right of each boxplot.

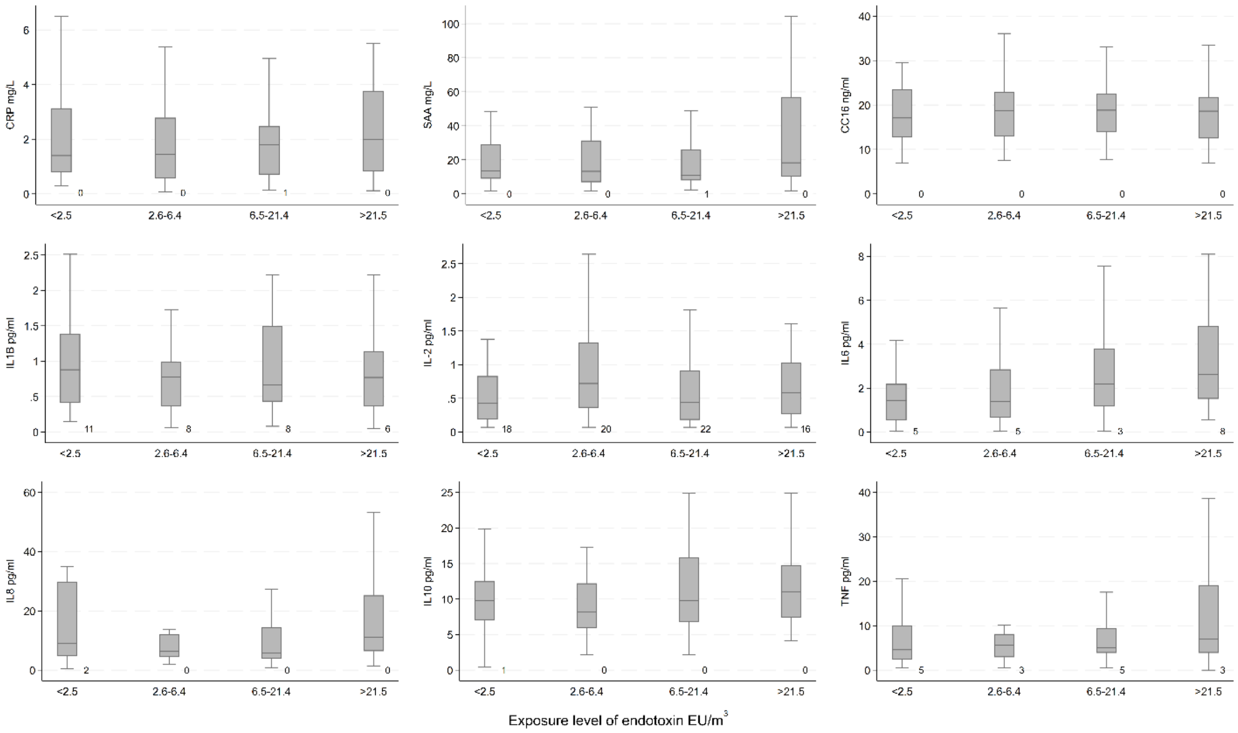

Figure S7 Unadjusted inflammatory marker concentrations by exposure level of endotoxin among recycling workers. Number of measurements below MDD at the bottom of each boxplot.

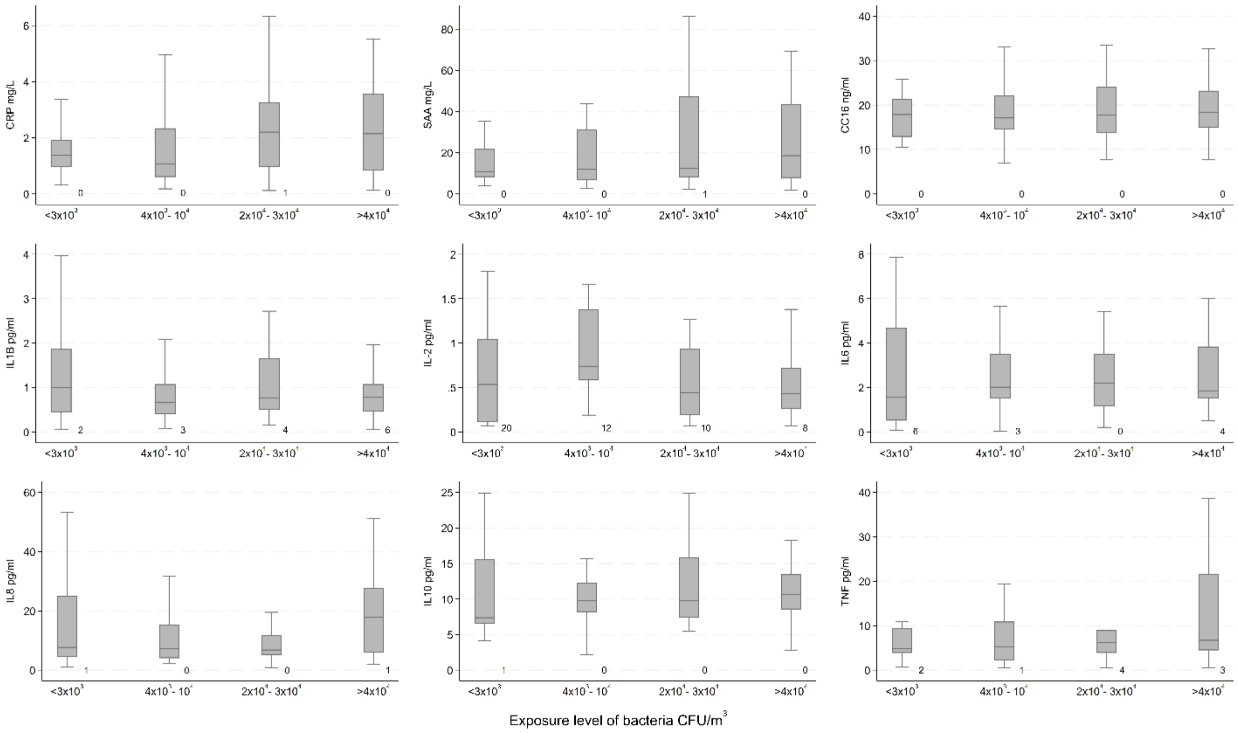

Figure S8 Unadjusted inflammatory marker concentrations by exposure level of bacteria among recycling workers. Number of measurements below MDD at the bottom of each boxplot.

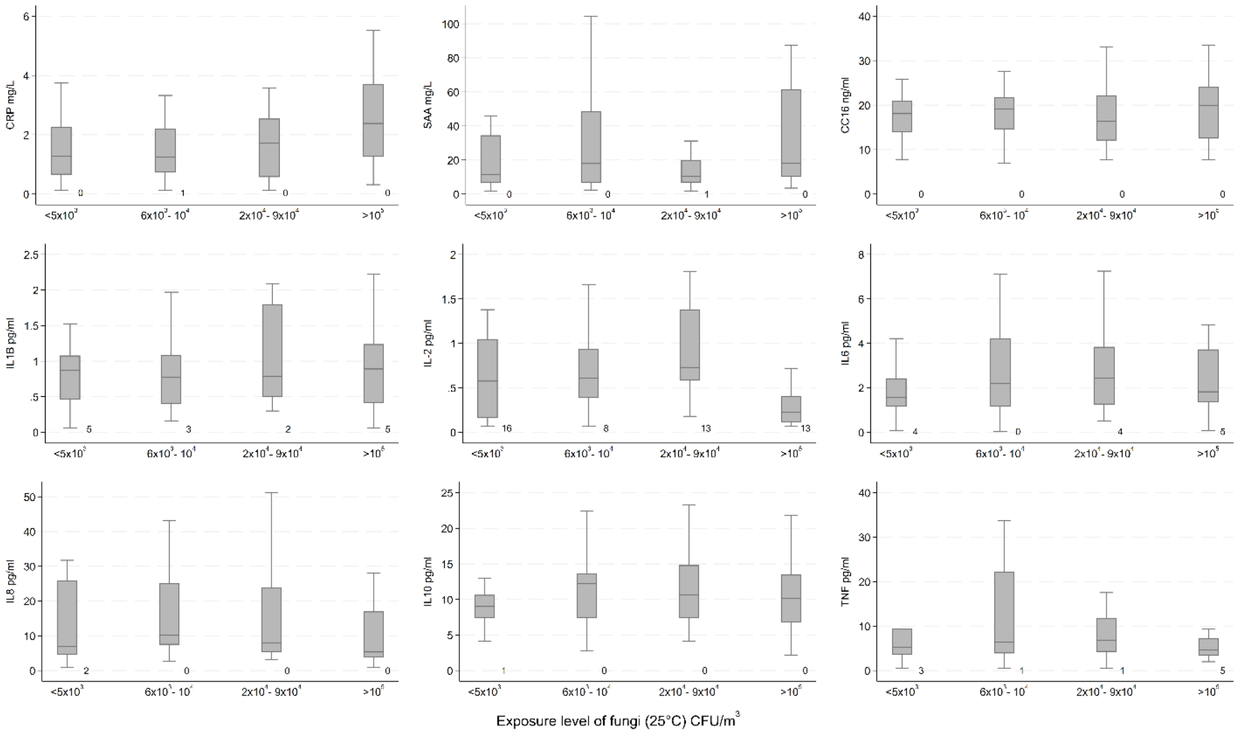

Figure S9 Unadjusted inflammatory marker concentrations by exposure level of fungi (25 °C) among recycling workers. Number of measurements below MDD at the bottom of each boxplot.

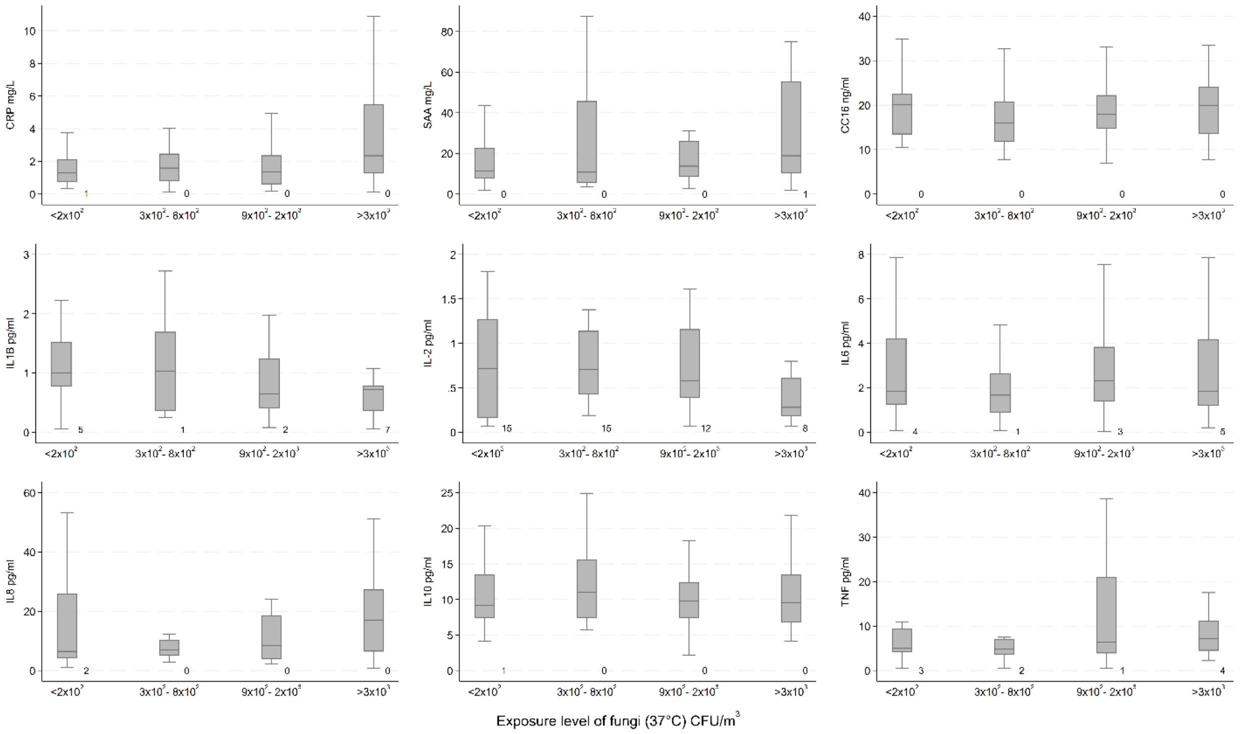

Figure S10 Unadjusted inflammatory marker concentrations by exposure level of fungi (37 °C) among recycling workers. Number of measurements below MDD at the bottom of each boxplot.

1. Eurostat. Municipal waste statistics 2023 [updated 08-22-2023. Available from: https://ec.europa.eu/eurostat/statistics-explained/index.php?title=Municipal_waste_statistics#Municipal_waste_generation.
2. European Commission. A European Green Deal 2019 [Available from: https://commission.europa.eu/strategy-and-policy/priorities-2019-2024/european-green-deal_en.
3. Statistics Denmark. Employeed in the waste and recycling industry from 2008-2022 (in Danish) 2022 [Available from: https://www.statistikbanken.dk/RAS309.
4. Douwes J, Eduard W, Thorne PS. Bioaerosols. In: Heggenhougen HK, editor. International Encyclopedia of Public Health. Oxford: Academic Press; 2008. p. 287-97.
5. Liebers V, Raulf-Heimsoth M, Bruning T. Health effects due to endotoxin inhalation (review). Arch Toxicol. 2008;82(4):203-10.
6. Athanasiou M, Makrynos G, Dounias G. Respiratory health of municipal solid waste workers. Occup Med (Lond). 2010;60(8):618-23.
7. Bunger J, Schappler-Scheele B, Hilgers R, Hallier E. A 5-year follow-up study on respiratory disorders and lung function in workers exposed to organic dust from composting plants. Int Arch Occup Environ Health. 2007;80(4):306-12.
8. Madsen AM, Raulf M, Duquenne P, Graff P, Cyprowski M, Beswick A, et al. Review of biological risks associated with the collection of municipal wastes. Sci Total Environ. 2021;791:148287.
9. Sigsgaard T, Abel A, Donbaek L, Malmros P. Lung function changes among recycling workers exposed to organic dust. Am J Ind Med. 1994;25(1):69-72.
10. Rylander R, Thorn J, Attefors R. Airways inflammation among workers in a paper industry. Eur Respir J. 1999;13(5):1151-7.

11. Sigsgaard T, Jensen LD, Abell A, Wurtz H, Thomsen G. Endotoxins isolated from the air of a Danish paper mill and the relation to change in lung function: an 11-year follow-up. Am J Ind Med. 2004;46(4):327-32.
12. Timm M, Madsen AM, Hansen JV, Moesby L, Hansen EW. Assessment of the total inflammatory potential of bioaerosols by using a granulocyte assay. Appl Environ Microbiol. 2009;75(24):7655-62.
13. Poole CJM, Basu S. Systematic Review: Occupational illness in the waste and recycling sector. Occup Med (Lond). 2017;67(8):626-36.
14. Wouters IM, Hilhorst SK, Kleppe P, Doekes G, Douwes J, Peretz C, Heederik D. Upper airway inflammation and respiratory symptoms in domestic waste collectors. Occup Environ Med. 2002;59(2):106-12.
15. Rasmussen PU, Frederiksen MW, Caroe TK, Madsen AM. Health symptoms, inflammation, and bioaerosol exposure in workers at biowaste pretreatment plants. Waste Manag. 2023;167:173-82.
16. Legal Information. Executive Order on Waste (in Danish) 2021 [Available from: https://www.retsinformation.dk/eli/lta/2021/2512.
17. Ministry of Environment of Denmark. Action Plan for Circular Economy. 2021.
18. Hansen KK, Schlunssen V, Broberg K, Ostergaard K, Frederiksen MW, Madsen AM, Kolstad HA. Exposure levels of dust, endotoxin, and microorganisms in the Danish recycling industry. Ann Work Expo Health. 2023;67(7):816-30.
19. Stanojevic S, Kaminsky DA, Miller MR, Thompson B, Aliverti A, Barjaktarevic I, et al. ERS/ATS technical standard on interpretive strategies for routine lung function tests. Eur Respir J. 2022;60(1).
20. Hansen MRH, Jors E, Sandbaek A, Sekabojja D, Ssempebwa JC, Mubeezi R, et al. Organophosphate and carbamate insecticide exposure is related to lung function change among smallholder farmers: a prospective study. Thorax. 2021;76(8):780-9.
21. Basinas I, Sigsgaard T, Heederik D, Takai H, Omland O, Andersen NT, et al. Exposure to inhalable dust and endotoxin among Danish livestock farmers: results from the SUS cohort study. J Environ Monit. 2012;14(2):604-14.
22. Whitcomb BW, Schisterman EF. Assays with lower detection limits: implications for epidemiological investigations. Paediatr Perinat Epidemiol. 2008;22(6):597-602.
23. StataCorp. Stata Statistical Software: Release 17. College Station, TX: StataCorp LLC; 2021.
24. Hughes JP. Mixed effects models with censored data with application to HIV RNA levels. Biometrics. 1999;55(2):625-9.
25. Rudnicka AR, Rumley A, Lowe GD, Strachan DP. Diurnal, seasonal, and blood-processing patterns in levels of circulating fibrinogen, fibrin D-dimer, C-reactive protein, tissue plasminogen activator, and von Willebrand factor in a 45-year-old population. Circulation. 2007;115(8):996-1003.
26. Ellulu MS, Patimah I, Khaza'ai H, Rahmat A, Abed Y. Obesity and inflammation: the linking mechanism and the complications. Arch Med Sci. 2017;13(4):851-63.
27. Bui DS, Lodge CJ, Burgess JA, Lowe AJ, Perret J, Bui MQ, et al. Childhood predictors of lung function trajectories and future COPD risk: a prospective cohort study from the first to the sixth decade of life. Lancet Respir Med. 2018;6(7):535-44.
28. Heldal KK, Halstensen AS, Thorn J, Eduard W, Halstensen TS. Airway inflammation in waste handlers exposed to bioaerosols assessed by induced sputum. Eur Respir J. 2003;21(4):641-5.
29. Madsen AM, Thilsing T, Baelum J, Garde AH, Vogel U. Occupational exposure levels of bioaerosol components are associated with serum levels of the acute phase protein Serum Amyloid A in greenhouse workers. Environ Health. 2016;15:9.
30. Eduard W, Pearce N, Douwes J. Chronic bronchitis, COPD, and lung function in farmers: the role of biological agents. Chest. 2009;136(3):716-25.
31. Heldal KK, Madso L, Huser PO, Eduard W. Exposure, symptoms and airway inflammation among sewage workers. Ann Agric Environ Med. 2010;17(2):263-8.

32. Schlunssen V, Madsen AM, Skov S, Sigsgaard T. Does the use of biofuels affect respiratory health among male Danish energy plant workers? Occup Environ Med. 2011;68(7):467-73.
33. Roponen M, Seuri M, Nevalainen A, Hirvonen MR. Fungal spores as such do not cause nasal inflammation in mold exposure. Inhal Toxicol. 2002;14(5):541-9.
34. Farokhi A, Heederik D, Smit LAM. Respiratory health effects of exposure to low levels of airborne endotoxin - a systematic review. Environ Health. 2018;17(1):14.
35. Bolund AC, Miller MR, Sigsgaard T, Schlunssen V. The effect of organic dust exposure on long-term change in lung function: a systematic review and meta-analysis. Occup Environ Med. 2017;74(7):531-42.
36. Walser SM, Gerstner DG, Brenner B, Bunger J, Eikmann T, Janssen B, et al. Evaluation of exposure-response relationships for health effects of microbial bioaerosols - A systematic review. Int J Hyg Environ Health. 2015;218(7):577-89.
37. Zamfir M, Gerstner DG, Walser SM, Bunger J, Eikmann T, Heinze S, et al. A systematic review of experimental animal studies on microbial bioaerosols: Dose-response data for the derivation of exposure limits. Int J Hyg Environ Health. 2019;222(2):249-59.
38. Hill AB. The Environment and Disease: Association or Causation? Proc R Soc Med. 1965;58(5):295-300.
39. Borsboom GJ, van Pelt W, van Houwelingen HC, van Vianen BG, Schouten JP, Quanjer PH. Diurnal variation in lung function in subgroups from two Dutch populations: consequences for longitudinal analysis. Am J Respir Crit Care Med. 1999;159(4 Pt 1):1163-71.
40. Pepys MB, Hirschfield GM. C-reactive protein: a critical update. J Clin Invest. 2003;111(12):1805-12.
41. Tape C, Kisilevsky R. Apolipoprotein A-I and apolipoprotein SAA half-lives during acute inflammation and amyloidogenesis. Biochim Biophys Acta. 1990;1043(3):295-300.
42. Broeckaert F, Clippe A, Knoops B, Hermans C, Bernard A. Clara cell secretory protein (CC16): features as a peripheral lung biomarker. Ann N Y Acad Sci. 2000;923:68-77.
43. Donohue JH, Rosenberg SA. The fate of interleukin-2 after in vivo administration. J Immunol. 1983;130(5):2203-8.
44. Waage A, Brandtzaeg P, Halstensen A, Kierulf P, Espevik T. The complex pattern of cytokines in serum from patients with meningococcal septic shock. Association between interleukin 6, interleukin 1, and fatal outcome. J Exp Med. 1989;169(1):333-8.
45. Huhn RD, Radwanski E, Gallo J, Affrime MB, Sabo R, Gonyo G, et al. Pharmacodynamics of subcutaneous recombinant human interleukin-10 in healthy volunteers. Clin Pharmacol Ther. 1997;62(2):171-80.
46. Liu C, Chu D, Kalantar-Zadeh K, George J, Young HA, Liu G. Cytokines: From Clinical Significance to Quantification. Adv Sci (Weinh). 2021;8(15):e2004433.
